# Factors influencing deprescribing in community-dwelling patients with palliative needs in primary care: A qualitative systematic review

**DOI:** 10.1177/02692163261465769

**Published:** 2026-07-31

**Authors:** Jesse F. van Weelderen, Ankie C. M. Hazen, Alexa M. M. Mulder, Eric C. T. Geijteman, Saskia C. C. M. Teunissen, Dorien L. M. Zwart, Matthew P. Grant

**Affiliations:** 1 Centre of Expertise in Palliative Care Utrecht, Julius Centre for Health Sciences and Primary Care, Department of General Practice & Nursing Science, University Medical Centre Utrecht, Utrecht, The Netherlands; 2 Department of Medical Oncology, Erasmus Medical Centre Cancer Institute, Rotterdam, The Netherlands

**Keywords:** deprescriptions, inappropriate prescribing, polypharmacy, primary health care, palliative care, systematic review

## Abstract

**Background:**

Patients with palliative needs often use potentially inappropriate medications, leading to adverse health outcomes. Deprescribing remains poorly enacted in primary care. Understanding the factors influencing deprescribing is essential for successful implementation into clinical practice.

**Aim:**

To explore factors influencing deprescribing in community-dwelling patients with palliative needs in primary care from the perspectives of patients, their caregivers and primary healthcare practitioners.

**Design:**

Qualitative systematic review using thematic synthesis, registered in PROSPERO (CRD42024620219).

**Data sources:**

MEDLINE, Embase, CINAHL, and PsycINFO were searched from 2000 to January 2025, directed by the Palliative cAre Literature rEview iTeraTive mEthod (PALETTE) framework. Studies were included if they reported on independently living adults with palliative care needs.

**Results:**

23 studies were included. Four themes were established: (i) initial stance towards deprescribing: shaped by values, general perceptions of (de)prescribing, and competencies. This served as the premise for (ii) the deprescribing conversation: characterised by an individualised deliberation, shared decision-making, and tailored communication and practical strategies. During this conversation, (de)prescribing was reconsidered in the context of the individual patient, informing final decisions. Whether and how these conversations occurred was dependent on (iii) organisational factors, including systematic work processes and interprofessional collaboration. All factors operated within and interacted with (iv) the context of care, containing system-level prerequisites.

**Conclusions:**

Deprescribing in community-dwelling patients with palliative needs in primary care can be optimised by employing a multidimensional strategy that includes patient-centredness, shared decision-making, education, collaboration within the multidisciplinary care team, and systematic work processes, ultimately aiming to improve quality of life.

**What is already known about the topic**

- Patients with palliative needs frequently use potentially inappropriate medications, associated with adverse health outcomes.
- Deprescribing is not routinely or systematically performed in primary care.
- Factors influencing deprescribing in community-dwelling patients with palliative needs in primary care have not yet been compiled in the literature.

**What this paper adds**

- Deprescribing is perceived by primary healthcare practitioners as risky, time-consuming and complex, related to clinical uncertainties, insufficient training, and limited (awareness of) clinical guidance.
- Deprescribing conversations rely on individualised patient assessments, shared decision-making, and tailored communication and practical strategies.
- Organisational facilitators include the establishment of structured work processes, clear task delineation, and interprofessional collaboration.

**Implications for practice, theory, or policy**

- Education and training for primary healthcare practitioners and patients may reduce negative perceptions and support the prioritisation of deprescribing.
- Deprescribing conversations can be optimised by considering the patient’s needs and goals of care, and utilising shared decision-making.
- The multidisciplinary primary care team should establish systematic work processes adapted to local contexts, ensuring sustainable impact.

## Introduction

Patients with palliative needs use an average of eleven medications in the last year of life.^1^ Although treatment goals in palliative care shift from curative intent to optimising quality of life and symptom control,^2^ over 80% of patients at the end of life use at least one potentially inappropriate medication, with 30% continuing until death.^3^ Medications may become potentially inappropriate when: (i) potential harms outweigh anticipated benefits; (ii) time to benefit exceeds life expectancy; or (iii) continued use conflicts with goals of care.^4^ Common potentially inappropriate medications in the palliative setting include preventive medications such as lipid-lowering drugs, antihypertensives, and osteoporosis medications.^5^ The extensive use of potentially inappropriate medications constitutes a significant concern,^6^ as patients with palliative needs are particularly susceptible to adverse drug events due to their heightened physiological vulnerability, arising from progressive life-limiting illness, bodily changes that accompany end-stage disease, and multimorbidity.^7–10^ In this population, polypharmacy is also associated with drug-drug interactions, reduced quality of life, and elevated healthcare costs.^6,9,10^ Adverse health outcomes of inappropriate polypharmacy are preventable through the act of deprescribing: *“the process of withdrawal of an inappropriate medication, supervised by a health care professional with the goal of managing polypharmacy and improving outcomes.”*^11^ Deprescribing interventions in palliative care have shown to enhance medication appropriateness, reduce negative health outcomes, and lower costs.^12^

Patients may experience palliative needs when they encounter physical, psychological, social, or spiritual challenges related to life-threatening illness, indicating a potential benefit from palliative care.^13^ For this article, we focus on patients with a limited life expectancy, such as those with advanced disease. In numerous countries, community-dwelling patients with palliative needs are primarily managed and prescribed medications through primary care teams. However, deprescribing remains poorly enacted in routine primary care practice. When discontinuation of potentially inappropriate medications occurs, it is typically late, within the last 2 months of life.^14^ Numerous context-specific factors influencing the enactment of deprescribing have been described in previous reviews, which focused on elderly in primary care or patients with palliative needs across multiple settings.^15–20^ The perspectives of stakeholders involved in deprescribing in community-dwelling patients with palliative needs in primary care have not been specifically synthesised in the literature, limiting the successful implementation of deprescribing interventions.^21^

This qualitative systematic review aims to explore factors influencing deprescribing in community-dwelling patients with palliative needs, from the perspectives of patients, their caregivers, and primary healthcare practitioners.

## Methods

This qualitative systematic review using thematic synthesis^22^ was reported in accordance with the Preferred Reporting Items for Systematic Reviews and Meta-Analyses (PRISMA) and the Enhancing transparency in reporting the synthesis of qualitative research (ENTREQ) statement.^23–25^ We adopted a constructivist paradigm, which assumes multiple subjective realities exist and are socially constructed based on individual experiences.^26^ The protocol was prospectively registered in PROSPERO (CRD42024620219), accessible at: https://www.crd.york.ac.uk/PROSPERO/view/CRD42024620219.

### Eligibility criteria

The eligibility criteria for study selection are specified in Table 1.

**Table 1.** Eligibility criteria for study selection.

Inclusion criteria	Exclusion criteria
1. Population – adult patients with palliative needs, defined as patients having advanced disease (e.g. metastatic cancer, advanced heart failure, COPD or dementia, or dialysis patients not eligible for transplantation), receiving palliative care, or mentioned to have limited life expectancy, to be terminally ill or at the end-of-life.^a^ 2. Phenomena of interest – describing factors influencing deprescribing in general or through any type of deprescribing intervention, regardless of type of medication. 3. Setting – community-dwelling patients under the (presumed) care of the general practitioner. Community-dwelling was defined as living independently at home or in extra care housing (including home hospice).^a^ 4. Design – absolute or partial qualitative design, as this was considered most appropriate for exploring influencing factors. 5. Peer-reviewed, empirical, English or Dutch articles.	1. Population – patients <18 years. 2. Setting – nursing homes, long-term residential care facilities, or inpatient hospices, in which patients are dependent on daily, constant nursing care. Increased patient monitoring within these facilities naturally promotes deprescribing opportunity and awareness, and transitions to these facilities create additional deprescribing opportunities, while also changing the nature of deprescribing from proactive to reactive. 3. Design – quantitative methodology. 4. Publication type – reviews, conference abstracts or proceedings, dissertations, and opinion papers.

COPD: chronic obstructive pulmonary disease.

a Mixed-population or mixed-setting studies were eligible if they included any community-dwelling patient with palliative needs under the (presumed) care of the general practitioner, or any caregiver or primary healthcare professional involved in their care.

### Search methods

The review question was defined in advance and remained unchanged throughout the review process. The search strategy was developed using the Palliative cAre Literature rEview iTeraTive mEthod (PALETTE) framework,^27^ an iterative method suitable for reviews in palliative care and qualitative research. In accordance with this framework, co-authors with expertise in deprescribing research were consulted to identify relevant literature, informing selection of initial key articles (i.e. articles that unequivocally meet the inclusion criteria) and development of the search strategy. No additional external experts were consulted. The search string combined terms for the (i) population: patients with palliative needs; (ii) phenomena of interest: deprescribing; (iii) setting: community-dwelling OR primary care;^28^ and (iv) design: qualitative. It was supplemented by keywords and validated search terms.^29–32^ Text mining tools were not used to support term identification. The search strategy was optimised in consultation with an experienced medical librarian. Following the PALETTE framework, key articles were iteratively identified and used to refine and validate the search strategy. Reference lists and citations of included articles and related reviews were hand-searched to identify additional eligible literature, informing further refinement of the search strategy. The full search strategy for MEDLINE is detailed in Supplemental Table 1. Publications were searched from 2000 until January 2025, as the term “deprescribing” first appeared in the literature in the early 2000.^33^ The final search was conducted in MEDLINE, Embase, CINAHL, and PsycINFO on the 5th of January 2025. Grey literature was not searched, as we considered it unlikely that this would have substantially altered the emerging synthesis given our comprehensive database search.^34^

### Data collection and analysis

#### Selection of studies

All references were imported into Endnote to remove duplicates, then transferred to Rayyan for screening. Titles and abstracts were independently screened by two researchers (JW and AM), blinded to each other’s decisions, with a third researcher (MG) reviewing a 10% sample. Disagreements were resolved through discussion. The same procedure was applied to full-text screening. AH and MG reviewed all eligible studies to determine final inclusion.

#### Data extraction

Study characteristics, including year of publication, country, aims, design, data collection, participants, patient population, setting, recruitment strategy, data analysis, and definitions, were extracted using a predetermined form. JW and AM independently performed data extraction, with AH and MG reviewing a sample. Consensus was reached through dialogue.

#### Quality assessment

JW and AM independently appraised study quality, using the Critical Appraisal Skills Programme (CASP) Qualitative Studies Checklist^35^ for qualitative studies and the Mixed Methods Appraisal Tool (MMAT)^36^ for mixed-methods studies. Discrepancies were resolved following discussion. Numerical scores were calculated to facilitate transparent comparison across studies, with scores below 6 pragmatically classified as indicative of lower quality. Studies were retained regardless of appraisal outcome, but quality assessment informed interpretation and reporting of results. We verified whether factors identified in lower quality studies were also supported by studies deemed of appropriate quality.

#### Data analysis

Data were analysed using inductive thematic synthesis,^22^ as it enables exploration of influencing factors, and is suitable for practice-oriented topics like deprescribing. Analysis comprised three stages: (1) line-by-line coding of qualitative findings in the results sections, applying the constant comparative method, with NVivo15 software. Excerpts not meeting inclusion criteria (e.g. regarding non-palliative or secondary care) were excluded from analysis. Excerpts of uncertain eligibility were retained if they could reasonably be interpreted as relevant to community-dwelling patients with palliative needs in primary care. A sensitivity analysis was conducted whereby these excerpts of uncertain eligibility were excluded, which did not materially affect the (sub)themes. Coding was performed independently by JW and AM, reaching consensus through discussion; (2) organising codes into subthemes; and (3) synthesising subthemes into themes through further interpretative analysis. Preliminary subthemes and themes were developed collaboratively by JW, AH, AM, and MG, and finalised in consultation with all authors. As many studies combined perspectives from different healthcare disciplines, the term healthcare practitioners was used for these combined perspectives. Separate views were reported when discernible.

## Results

**Figure 1.**
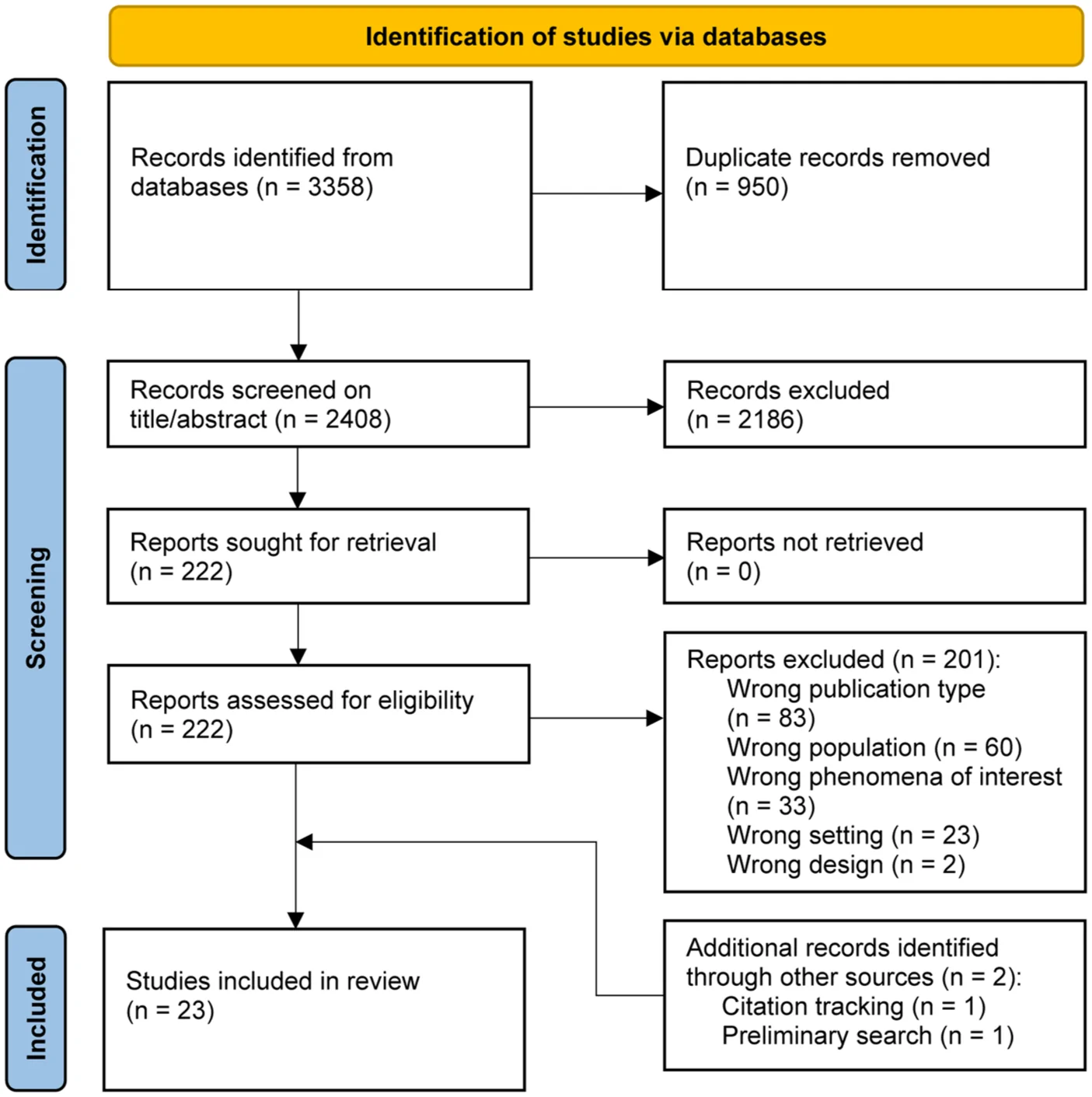
PRISMA flow diagram of study selection process.

The database searches yielded 3358 records, of which 2408 remained after duplicate removal. Following title and abstract screening, 2186 records were excluded. 222 full-text articles were assessed for eligibility. 23 articles met the inclusion criteria (Figure 1). A full list of excluded articles based on full-text screening, with reasons for exclusion, is provided in Supplemental Table 4.

### Study characteristics

Three studies were conducted in Australia,^37–39^ one in Belgium,^3^ one in Denmark,^40^ three in Northern Ireland,^41–43^ one in Norway,^44^ seven in the Netherlands,^45–51^ five in the United Kingdom,^52–56^ and two in the United States.^57,58^ Years of publication ranged from 2013 to 2024. Four studies adopted a mixed-methods design,^3,43,47,49^ while the remaining studies utilised a qualitative design. Data were collected through interviews,^3,39,41,42,44–46,48–51,53–56,58^, focus groups,^38,40^ or a combination of both.^57^ Other data sources included chart reviews,^37,47^ an online discussion forum,^52^ and free-text survey responses.^43,49^ Seventeen studies included additional settings alongside community-based primary care, such as hospitals, inpatient hospices, and nursing homes.^38–43,45–50,53–55,57,58^ The perspectives of patients with palliative needs, their caregivers, general practitioners (GPs), medical specialists, palliative medicine consultants, advanced practice providers, nurses, pharmacists, health care assistants, and allied health professionals (dentistry, physiotherapy) were reported. Health care assistants either worked in home care or nursing homes, providing basic nursing care and personal support. Eighteen studies explored factors influencing deprescribing in general, while five studies focused on specific medications.^43,47,49,51,52^ A detailed summary of study characteristics is provided in Table 2.

**Table 2.** Summary of characteristics of included studies.

Study details (first author, publication year, country)	Aim(s)	Study design	Methods	Setting	Participants
Alwidyan, 2023, Northern Ireland^41^	(i) To explore perceptions of existing deprescribing practice from the perspectives of HCPs, (ii) explore barriers to and enablers of deprescribing in older patients at end-of-life in hospice care using the Theoretical Domains Framework as a “theoretical lens,” (iii) identify the relevant domains in this framework and map these to intervention components that could be delivered in a future intervention to target behaviour change	Qualitative	Semi-structured interviews	Hospices including inpatient, day therapy and community services	*N* = 20, of which 9 doctors, 9 nurses, 2 pharmacists. Work setting: 7 community services, 3 inpatient unit, 3 inpatient unit and community services, 2 all hospice settings, 2 inpatient unit and outpatient unit, 1 clinical director and inpatient unit, 1 outpatient unit and community services, 1 hospice and hospital
Cortis, 2017, Australia^37^	To gain insight into which types of community-based patients with palliative needs beneﬁt from medication management services in order to guide local service development	Qualitative	Analysis of a case series of medication reviews conducted by a palliative care pharmacist	Community	*N* = 15 patient cases
Dees, 2018, The Netherlands^45,a^	To gain insight into the perspectives of patients, close relatives, nurses, and physicians on medication management for patients with a life expectancy of less than 3 months	Qualitative	In-depth interviews	Community, nursing home, hospital (general or academic hospital), hospice	*N* = 76 (based on 18 patient cases), of which 17 patients, 12 relatives, 15 nurses, 20 clinical specialists (3 trainees), 12 GPs (1 trainee). Patient cases: place of residence: 8 at home, 1 nursing home, 6 hospice, 3 hospital
Geijteman, 2016, The Netherlands^46,a^	To identify factors contributing to the use of potentially inappropriate medications in the last stage of life from the perspectives of older adults and their relatives and physicians	Qualitative	Interviews	Community, nursing home, hospital (general or academic hospital), hospice	*N* = 61 (based on 18 patient cases), of which 17 patients, 12 relatives, 20 clinical specialists, 12 GPs
Hall, 2023, USA^57^	To identify clinicians and patient perspectives on factors related to deprescribing to inform design of a deprescribing program for dialysis clinics	Qualitative	Semi-structured interviews and focus groups	Primary care and dialysis clinics	*N* = 76, of which 53 clinicians (via 8 focus groups and 11 interviews) and 23 patients (all interviews). Clinicians: 24 dialysis (12 physicians, 3 nurses, and 9 advanced practice providers), 18 primary care (14 physicians, 4 advanced practice providers), and 11 pharmacists
Huisman, 2021, The Netherlands^49,a^	To identify the opinions of physicians about the use of anticoagulants at the end of life	Mixed-methods	Using quantitative and qualitative data from a vignette study and qualitative data from a secondary analysis of an interview study	Community, nursing home, hospital (general or academic hospital), hospice	Vignette study: *N* = 321, of which 174 GPs, 147 clinical specialists. Interviews: *N* = 23, of which 8 GPs, 15 clinical specialists
Huisman, 2020, The Netherlands^48,a^	To gain insight into the perspectives of patients, informal caregivers, nurses and physicians on the role of nurses in medication management at the end of life	Qualitative	Semi-structured interviews	Community, nursing home, hospital (general or academic hospital), hospice	*N* = 76 (based on 18 patient cases), of which 17 patients, 12 relatives, 15 nurses, 20 medical specialists, 12 GPs
Huisman, 2021, The Netherlands^47^	(i) To gain more insight into management of antithrombotics in patients with a life-limiting disease, (ii) to conduct an in-depth analysis of the (dis)continuation of antithrombotics in patients in the last three months of life	Chart review study	Secondary analysis of a retrospective chart review of medical records	Community, hospital, hospice	*N* = 108 charts. Place of residence: 36 community, 23 hospice, 49 hospital
Kuruvilla, 2018, Australia^38^	(i) To identify perceived gaps and challenges to medication management within community palliative care (CPC) from the perspectives of both consumers and health care professionals, (ii) to assess stakeholder opinion as to the benefits and logistics of integrating a clinical pharmacist into a CPC service in addressing some of those gaps	Qualitative	Focus groups	Community palliative care service providing community-based palliative care	*N* = 20 (via 3 focus groups, 2 for HCPs and 1 for palliative care consumers), of which 12 HCPs, 2 patients, 6 caregivers. HCP group: 3 palliative care medical consultants, 1 palliative care medical registrar, 1 palliative care nurse practitioner, 1 community nurse, 1 hospital pharmacist, 1 community pharmacist. Consumer group: 2 patients, 6 caregivers
Lundby, 2019, Denmark^40^	To explore different HCPs’ perspectives on deprescribing in older patients with limited life expectancy	Qualitative	Semi-structured focus groups	Primary and secondary care	*N* = 32 (via 6 focus groups: 1 FPs, 1 Geriatricians, 1 Clinical pharmacologists (secondary care), 1 Clinical pharmacists (secondary care), 1 nurses (primary and secondary care), 1 health care assistants (primary care)), of which 5 FPs, 5 Geriatricians (3 trainees), 5 Clinical pharmacologists (1 trainee), 6 Clinical pharmacists, 6 nurses (3 working in primary care and 3 in secondary care) and 5 health care assistants
McCloskey, 2018, Northern Ireland^42^	To explore proxy decision makers’ expectations of prescribed medications for people with advanced dementia and to consider how these change with changing goals of care and dementia progression	Qualitative	Semi-structured interviews	Community and nursing home	*N* = 15 proxy decision-makers. Place of care of patient: 10 community, 5 nursing home
Mc Namara, 2017, Australia^39^	To explore current approaches to multimorbidity management, and perceived barriers and enablers to deliver appropriate medication management for community-dwelling patients with multimorbidity and polypharmacy, from a broad range of healthcare professional perspectives in Australia	Qualitative	Semi-structured interviews	Primary, secondary and tertiary care	*N* = 26, of which 5 GPs, 3 general medicine consultants, 2 geriatricians, 1 clinical pharmacologist, 2 practice nurses, 2 nurse practitioners, 2 hospital nurses, 4 hospital pharmacists, 2 community pharmacists, 1 dentist, 1 dental hygienist, 1 physiotherapist. Setting: 12 community, 14 hospital
Parsons, 2019, UK^52^	To investigate the experiences and perspectives of carers and family members when antidementia medications (cholinesterase inhibitors and/or memantine) are stopped, by analysing archived threads and posts of an online discussion forum for people affected by dementia	Qualitative	Qualitative analysis of an online discussion forum	Not specified	Posts from 112 users. No information available on users
Parsons, 2013, Northern Ireland and the Republic of Ireland^43^	To evaluate the extent to which patient-related factors and physicians’ country of practice (Northern Ireland and the Republic of Ireland) influenced decision making regarding medication use in patients with end-stage dementia	Mixed-methods	Survey design comprising four vignettes to evaluate initiating/withholding or continuing/discontinuing specific medications (antibiotics, acetylcholinesterase inhibitors, memantine, statins and antipsychotics). Qualitative analysis of free text responses (reasons for recommending changes)	Community, nursing home and hospital	*N* = 662, of which 576 GPs and 67 hospital physicians
Pype, 2017, Belgium^3^	(i) Describing the medication use and use of potentially inappropriate medications in primary care at the end of life, (ii) exploring the barriers for GPs to deprescribing with their patients with palliative needs	Mixed-methods	Retrospective chart review and semi-structured interviews	Community	Chart review: *N* = 210. Interviews: *N* = 11 GPs
Robinson-Barella, 2014, UK^53^	To explore the challenges of, and potential solutions to, making decisions about deprescribing in a palliative care context	Qualitative	Semi-structured interviews	Primary and secondary care	*N* = 20, of which 3 GPs, 7 medical consultants (within various settings), 6 nurses (primary and secondary care), 4 specialist pharmacists
Sand, 2018, Norway^44^	To explore patients’ experiences of using medicines when they are living with far-advanced cancer and short life expectancy	Qualitative	Interviews	Daycare centre at a palliative clinic, patients living in the community	*N* = 15 patients
Sheard, 2012, UK^54^	To explore the barriers for doctors in the UK when diagnosing and treating advanced cancer patients with VTE	Qualitative	Interviews	Primary, secondary and tertiary care, including general practices, hospitals and hospices	*N* = 45, of which 10 GPs, 20 oncologists and 15 palliative medicine doctors (mixture of senior and junior staff)
Tjia, 2020, USA^58^	To describe nurses’ perspectives about their role in hospice family caregiver medication management and support, including medication review and deprescribing	Qualitative	Interviews	Primary care, home and inpatient hospice	*N* = 10 nurses, of which 6 home hospice, 3 inpatient hospice, 1 medical home coordinator for a primary care practice
Todd, 2016, UK^55^	To explore the lived experience of patients, carers and healthcare professionals in the context of medication use in life-limiting illness	Qualitative	Interviews using photo elicitation	Daycare centre at a specialist palliative care unit within an outpatient setting	*N* = 36, of which 12 patients, 12 carers, 6 GPs, 3 healthcare palliative medicine consultants, 3 advanced nurse practitioners
Van der Waal, 2024, The Netherlands^50^	To detail factors that influence deprescribing in the last phase of life, as identified by health care professionals working in primary care, patients and their caregivers	Qualitative	Semi-structured interviews	Primary care settings, including general practices, hospices and community palliative care teams	*N* = 17, of which 4 patients, 1 caretaker, 4 GPs, 3 pharmacists, 3 practice nurses, 1 nurse specialist, 1 community nurse
Van Middelaar, 2018, The Netherlands^51^	(i) To explore GPs’ routines and considerations on (de)prescribing antihypertensive medication in older patients, (ii) their judgement on usability of the current guideline, and (iii) needs for future support in this decision-making process	Qualitative	Semi-structured interviews	General practice	*N* = 15 GPs
Wyatt, 2022, UK^56^	(i) To explore GPs’ experiences of providing end-of-life care for people with cancer in the home setting and their perceptions of confidence in this role, (ii) to explore the role of GPs in delivering end-of-life care and understanding implications this has on policy design	Qualitative	Semi-structured interviews	Community	*N* = 19 GPs (7 trainees)

CPC: community palliative care; FP: family physician; GP: general practitioner; HCP: healthcare practitioner; PIM: potentially inappropriate medication; UK: United Kingdom; USA: United States of America; VTE: venous thromboembolism.

a The (qualitative) results of these papers were based on the same participants.

### Quality assessment

Quality assessment scores ranged from 4 to 10 for the included qualitative studies and from 3.5 to 7.6 for the mixed-methods studies. Five studies were considered low quality.^37,43,47,54,58^ The quality assessment is detailed in Supplemental Tables 2 and 3.

### Thematic synthesis

Four themes were established: (i) initial stance towards deprescribing; (ii) the deprescribing conversation; (iii) organisational factors; and (iv) the context of care.

**Figure 2.**
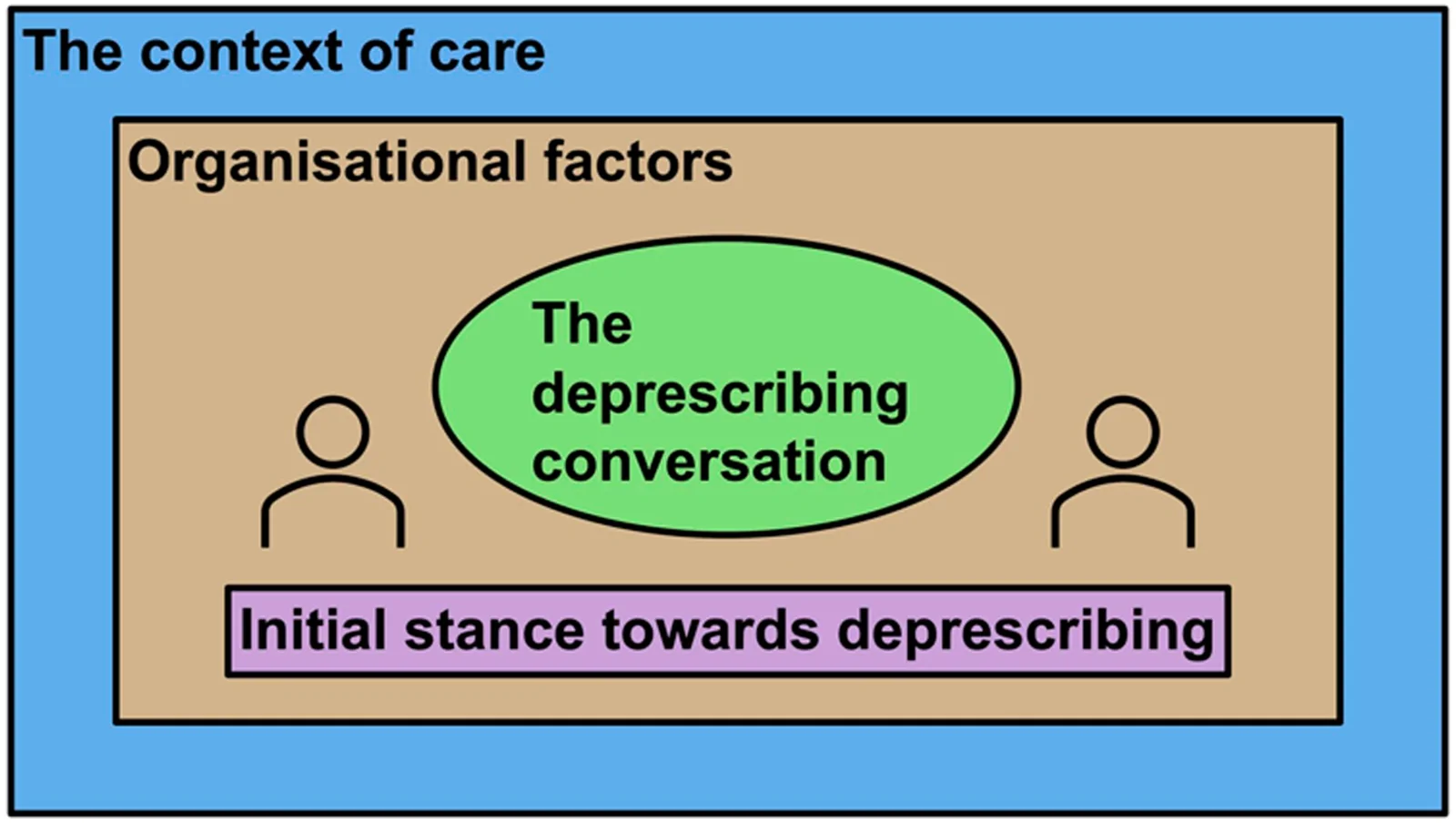
Schematic depiction of interrelationship of themes.

The initial stance towards deprescribing was shaped by values, general perceptions of (de)prescribing, and pharmacological and palliative care competencies, which influenced the willingness to consider deprescribing before engaging in such discussions. This stance served as the premise for the deprescribing conversation; the actual interaction between healthcare practitioners, patients, and caregivers in which (de)prescribing was reconsidered in the context of the individual patient, informing final decisions. Prominent factors influencing the conversation included the individualised deliberation of perceived benefits versus harms of deprescribing decisions, shared decision-making, and the communicative and practical strategies being employed. Whether and how these conversations occurred was dependent on organisational factors, including the establishment of systematic work processes and interprofessional collaboration. All factors operated within and interacted with (iv) the context of care, containing system-level prerequisites. The relationship of themes is schematically illustrated in Figure 2. Examples of data per (sub)theme are presented in Table 3.

**Table 3.** Examples of data from included studies per (sub)theme.

(Sub)themes	Examples of data^a^
**Theme 1: Initial stance towards deprescribing**	
Personal and professional values	Individuals and their relatives and physicians felt that potentially inappropriate medications should ideally be discontinued at the end of life.^46^ *“The physical beneficial consequences are that you could have a patient who has a better quality of life.”* (Ph3)^41^
General perceptions of medication use	Patients were ambivalent about taking medication: on the one hand, they knew very well they needed medication; on the other hand, they wanted deeply not to take it.^44^ *“The message we give as professionals is “once you’re on it, you’re on it forever.” I get why we do that for compliance, concordance . . . but maybe we need a bit of “this will be reviewed annually and we might change it” phrasing built in.”* (Participant 15)^3^
General perceptions of deprescribing	*“Anticoagulants were stopped in the hospice when she developed a thunderous CVA, resulting in hemiparesis and loss of speech. I get the idea of cessation, but it really sucks in case complications arise.”* GP 2^14^ *“If you stop something, it also makes people feel like, ‘Well apparently it’s no use anymore to treat me in the long term because . . .,’ and this confronts people with death, with ﬁniteness, I think.”* Family physician (FP15)^46^
Competencies	*“if they (medics) learnt about it as undergraduates, like anything, the more you do it, the easier it becomes and the more confident you feel in doing it”* (Participant 3)^53^
**Theme 2: The deprescribing conversation**	
Individualised clinical judgment	*“Yes, and then I let them eat their pills, that is, if it doesn’t outright harm them, if they do not have side effects, if they have a good quality of life, as you say yourself, then it isn’t at that point that I begin taking something from them.”* (FP 1)^40^ *“[. . .] So for example if you think someone may or may not have a DVT but they are otherwise quite poorly but comfortable, if they are not getting a lot of symptoms about that then it’s being overcautious about do I interfere with the rest of the quality of their life when they are not that symptomatic. And I think you can forget that they might just go and have a big PE and then they would be far more symptomatic. And then you think well if they are going to die relatively shortly, as long as they have a really big PE and go really quickly then it is not going to be too awful a thing. Your mind goes round all these things and then you think, well what if they just have a moderate sized PE and they are really breathless but don’t die”* (England, ID 8)^54^
The dialogue between healthcare practitioners, patients and caregivers	*“Collaborative. . . we should say (to the patient) “look, in a medical opinion, we could do this, but what do you think?” I think should be a proper open discussion”* (Participant 3).^53^ In particular, the importance of gently introducing deprescribing concepts that *“explain we’re not giving up on them”* and *“emphasising that we’re on a journey together and they’re not being left high and dry”* (Participant 9). [. . .] they flatten the hierarchy between the patient and themselves, in a bid to *“feel that they (patients) can ask things to me that maybe they otherwise won’t”* (Participant 10). In doing so, the participant felt the dynamic of the consultation was one of shared decision-making, rather than a *“traditional consultation”* with a paternalistic approach.^53^ *“I’ve have had a few conversations where people tell me; I don’t need it anymore. I have reached the age of 94. It’s been good, so why am I still taking 15 pills a day? Is this necessary? Then you do get interesting conversations. Indeed, what are we actually doing? Half the tablets you take are to prolong your life, and I can hear you very clearly saying that is something that isn’t a priority anymore.”* – Pharmacist 1^50^
Timing	*“Like it ends up being, if a patient is just really unwell. . . it’s probably then when [deprescribing] really starts being thought about”* (Participant 14, GP).^53^ *“Interviewer: Is this a good moment to get rid of several medications? Patient: Yes, the previous times it wasn’t an issue, and now it is. Interviewer: Imagine that the doctors had discussed such matters with you at an earlier stage, how would you have felt about it then? Patient: Yes, I ﬁnd that difﬁcult. No, I believe I would then have felt something like ‘Guys, do you want to get rid of me or something?’ [chuckles]”*^46^
**Theme 3: Organisational factors**	
Interprofessional collaboration	*“The hospital physicians believe that the FP should do it, and the FP thinks that the medical specialists at the hospital would probably make a decision about it.”* (Pharmacist 2)^40^ *“If we say ‘no, we will stop it’ and then they go to see a specialist who is happy to continue. Should we then contradict him and possibly cause trouble?”* (GP 10)^3^ *“Some patients haven’t seen their GPs regularly because they see specialists all the time, they might get scripts here and there from their specialists but not necessarily from their GP, the GP may not know of changes to medication. Whilst there’s sometimes good communication there isn’t always.”* (Palliative care nurse practitioner)^38^
Work processes, task division and supporting roles	*“I also think the process steps need to be better mapped out. Is the pharmacist involved, the GP, etc. All the steps need to be clear, who does what. Once you discuss with the patient and they agree to start the process, you are now confronted with actually not knowing how to deal with it. [. . .]”* – Practice nurse 1^50^ *“It’s a really important collaborator [nurses and health care assistants]. [. . .] Sometimes they pull themselves tight, the patients, when they visit us, and we see one picture of them. But seeing them in their own home, and the observations the nursing staff can provide us through this, that is extremely important.”* (FP 2)^40^
**Theme 4: The context of care**	
System-level factors	*“In real-life, what I notice, is that a consultation for a complaint is the trigger for a more careful inspection of medication use. . . Not that I do not dare to have the conversation, the opportunity is limited by a lack of time and space in the setting that you work in. . . A medication review can be complex, and the lack of urgency can result in that you let it go.”* – GP 3^50^ Healthcare professionals shared views around better establishing the culture of deprescribing within clinical practice. They highlighted perspectives around the importance of *“starting to think that way from the start”* (Participant 11) at the point of prescribing a medication.^53^ *“We had someone who broke her hip after a myocardial infarction (MI). She returned from hospital with an awful lot of medication, statins, metoprolol, everything. A cardiologist works with cardiology guidelines, if you have a MI; you qualify for the whole nine yards.”* – Nurse specialist 1^50^

CVA: cerebrovascular accident; DVT: deep vein thrombosis; FP: family physician; GP: general practitioner; MI: myocardial infarction; PE: pulmonary embolism; Ph: pharmacist; PIM: potentially inappropriate medication.

a Excerpts presented in italics and quotation marks represent direct participant quotations. The other excerpts reflect the authors’ interpretations.

### Theme 1: Initial stance towards deprescribing

This theme comprises four subthemes: (i) personal and professional values; (ii) general perceptions of medication use; (iii) general perceptions of deprescribing; and (iv) competencies.

#### Personal and professional values

Across healthcare practitioners, patients, and caregivers, there was a shared view that the use of unnecessary medications should be avoided, and a recognition of the value of deprescribing in palliative care.^3,38–43,45,46,50,51,53,55,57^ Participants generally expressed a willingness to engage in deprescribing,^38,41,55^ often being motivated by the desire to maintain or improve quality of life.^3,39–43,49^ Most patients preferred taking fewer medications.^37,38,41,44,50,57^

Healthcare practitioners acknowledged that patients’ perceptions of what constituted their quality of life varied. They noted that some patients valued prolonging life as much as possible and could view deprescribing as potentially reducing their quality of life.^40,49^ Other patients described valuing a focus on quality of life over quantity, which might lessen the perceived need for ongoing medication,^49,55^ though this did not always translate into patients’ readiness to discontinue medications.^37^

#### General perceptions of medication use

Medications were commonly perceived as a necessary evil; individuals recognised their clinical necessity yet acknowledged potential harms or burdens.^44,45^ Preventive medications were often regarded by healthcare practitioners as medically futile in the palliative care context, as their benefits were unlikely to be realised within the patient’s remaining lifespan.^3,40,43,47,49,54^ Patients held varying, sometimes conflicting beliefs regarding the benefits and risks of certain medications.^55^ Healthcare practitioners experienced that outdated beliefs, occasionally based on previous communications from healthcare practitioners, such as thinking certain medications were lifelong therapies, could cause patient and caregiver reluctance to deprescribing.^38,41,53,55,58^ Altering the phrasing used when prescribing new medications was reported to help align treatment expectations among healthcare practitioners, patients, and caregivers.^45,53,55^ Healthcare practitioners felt that certain beliefs, as well as long-term use or psychological reliance linked to symptom relief, could foster attachment to medications among patients and caregivers, and were correlated with stability.^38,49,50,57^ Participants expressed divergent perspectives regarding the place of financial matters in (de)prescribing decisions, ranging from definite to negligible.^41,45,50^

#### General perceptions of deprescribing

Both healthcare practitioners and patients assigned low priority to deprescribing, which, along with time limitations, led to greater focus on competing clinical issues.^3,46,50,57^ They considered continuation of medications safer than deprescribing.^50^ Healthcare practitioners expected deprescribing discussions to be challenging and time-consuming.^41,50,55,57,58^ Evidence-based clinical guidelines for deprescribing and managing multimorbid patients were scarce,^3,40,41,45,49,50^ contributing to healthcare practitioners’ heightened risk perceptions regarding deprescribing.^3,50^ Previous clinical experiences further shaped this risk perception, either negatively (e.g. by emergence of medication complications), or positively through the absence of adverse outcomes following deprescribing.^3,41,49,50^ Intrinsic motivation for deprescribing varied between physicians.^40,41^

Some healthcare practitioners and patients perceived deprescribing conversations as demoralising.^45,46,57^ Healthcare practitioners believed that initiating deprescribing discussions might confront patients and their caregivers with the prospect of impending death, evoke the feeling that their physician has given up on them, or raise concerns about not receiving optimal medical care.^3,46,51,53^ Some patients acknowledged experiencing such emotional responses.^46,53,55^ Certain healthcare practitioners also feared negative impacts on the patient relationship, conflict, or legal consequences, especially in the event of complications following deprescribing.^3,39,41^ For caregivers of patients with advanced dementia, decision-making was particularly distressing, involving feelings of fear of the future, guilt, and uncertainty regarding the appropriateness of their choices.^42,52^

#### Competencies

Healthcare practitioners described limitations in their pharmacological and palliative care competencies, including insufficient knowledge of which medications constitute potentially inappropriate medications, potential harms of their continued use, appropriate deprescribing approaches, and end-of-life conversation skills. These gaps were linked to limited education and professional experience.^41,45,49,53,57,58^ Among patients and caregivers, knowledge limitations were also reported alongside poor health literacy.^38,42,52,57^

### Theme 2: The deprescribing conversation

Three subthemes were delineated within this theme: (i) individualised clinical judgment; (ii) the dialogue between healthcare practitioners, patients and caregivers; and (iii) timing.

#### Individualised clinical judgment

Deprescribing decisions were typically informed through balancing the (perceived) harms against the (perceived) benefits on an individual basis during the deprescribing conversation, rather than relying on general perceptions.

Healthcare practitioners, patients, and caregivers regarded the perceived (lack of) clinical benefit of a drug, either in terms of symptom control or prevention of complications, as the central element in their individualised deliberation.^37,38,40,41,43,49,50,54,57^ This perceived benefit was dependent on multiple contextual and patient-specific factors, such as a patient’s life expectancy, stage of disease, and likelihood of clinical improvement. Healthcare practitioners held varying views on the appropriate life expectancy threshold or benchmarks for deprescribing, especially concerning anticoagulants.^49^ When prognosis was uncertain, healthcare practitioners were more hesitant to deprescribe.^39,41,45,49,50^ Prognostic estimations were informed by a variety of partly unvalidated predictors, including familial longevity, and prognostic instruments were not considered helpful in reducing prognostic uncertainty.^39^ The assessment of clinical benefit was also based upon the medication’s indication, and the risk and severity of adverse outcomes following deprescribing, in relation to type of medication and specific medical conditions or clinical situations.^3,39–43,45,46,49–55^

Healthcare practitioners, patients, and caregivers concurrently considered the potential advantages of deprescribing decisions for the individual, including the reduction of physical and psychological side effects, drug-drug interactions, and treatment burden.^3,40–42,47,55^ Healthcare practitioners noted that the patient’s quality of life prior to deprescribing formed part of the evaluation of potential benefit, as those with relatively high quality of life were expected to have less to gain.^3,37,39–44,47,49–51,53–55,57,58^ Another advantage was the prevention of potential future harms of continued medication use, such as falls, admissions related to side effects, prolonged inpatient stays, addiction, and loss of self.^37,41,44,47,49,57^ Potential future harms of continued medication use were also related to types of medication, medical history, and specific clinical situations.^41,49^

Healthcare practitioners reported difficulties in determining the ongoing indication and clinical effectiveness of certain treatments, particularly in case of poor medication adherence.^40^ Physicians highlighted that weighing the risks and benefits of continued anticoagulant use was especially challenging.^49,54^ Healthcare practitioners also noted that it could be difficult to distinguish between side effects and symptoms of underlying or new disease.^40^ A lack of clear evidence regarding both the clinical benefit and safety of deprescribing of certain drugs were mentioned to influence healthcare practitioners’ decision-making.^3,40,41,45,49^ In the absence of robust evidence and amid uncertainties, physicians often relied on common sense, clinical intuition, and professional experience to guide their decisions.^40,49^ Healthcare practitioners further believed deprescribing required a holistic patient assessment incorporating the perspectives, wishes and priorities of both patients and caregivers.^3,41,45,53,58^

Proxy decision-makers were guided by their understanding of the patient’s presumed wishes.^42^ In the setting of evident clinical decline in patients with advanced dementia, they perceived decreased benefits of medications, and were more open to deprescribing.^42^ Caregivers could be hesitant to stop certain medications that provided caregiver relief through the alleviation of symptoms, such as sleeplessness.^53,57^

#### The dialogue between healthcare practitioners, patients, and caregivers

A trusting relationship between healthcare practitioners, patients, and caregivers was regarded a critical facilitator of the deprescribing process.^45,50,53,57,58^ Both patients and healthcare practitioners advocated honesty and openness in deprescribing conversations,^3,46,53^ underpinned by the notion that patients should be aware of their palliative diagnosis in order to openly talk about appropriate medication use.^45^ Good communication was described to further improve deprescribing efforts, which included tailoring conversations to the individual patient and using communication strategies, such as focusing on the benefits of deprescribing.^41,45,46,53,55,58^

Healthcare practitioners, patients, and caregivers described that deprescribing should ideally involve shared decision-making.^3,40,45,49,53,55^ At the same time, patients and caregivers typically expressed trust in their physician’s expertise.^42,45,55,57^ Preferences regarding information provision varied among patients and caregivers: some sought detailed information to support their decision-making, whereas others primarily relied on their physician’s judgment.^42,57^ Caregivers were perceived by healthcare practitioners as important collaborators, particularly when patients were unwilling to discuss medications independently or when assessing the indication of specific treatments.^57^ Nonetheless, some healthcare practitioners considered patient and family involvement unnecessary or potentially harmful, partly given the complexity of deprescribing decisions.^45,49^ Various practical deprescribing strategies were discussed during consultations to enhance patient acceptance, including the consideration of alternate effective treatment options,^37,45,48,57^ temporary discontinuation of medications, which reassured patients by allowing the possibility of treatment reinitiation and assessment of drug efficacy,^40,41,52,53^ gradual tapering rather than abrupt cessation,^3,41,52^ discontinuing one medication at a time,^40^ and enlisting patients in symptom monitoring.^3^

Healthcare practitioners, patients, and caregivers acknowledged that deprescribing was more likely to occur if conversations were initiated by patients or caregivers themselves.^40,42,47,50^ Patient education and empowerment were identified by healthcare practitioners as potential facilitators of the deprescribing process.^41,53,57^

#### Timing

GPs reported deprescribing conversations to be initiated too late in the clinical trajectory.^53^ Limited awareness of deprescribing among healthcare practitioners often resulted in its practice being reactive in response to either complaints, side-effects, clinical deterioration, swallowing or digestion issues, or care transitions.^3,37,40–42,45–47,49–51,53,58^ Timing was considered appropriate when limited life expectancy could be expected, with deprescribing conversations ideally initiated proactively and early enough, yet not prematurely,^41,42,46,53^ preferably occurring outside of acute health episodes,^57^ and guided by individual care needs.^53^ Nonetheless, no consensus between healthcare practitioners was reached on optimal timing.^53^ Healthcare practitioners described that revisiting deprescribing conversations can be valuable, acknowledging that patients’ perspectives and clinical circumstances may change.^41,53^

### Theme 3: Organisational factors

This theme encompasses factors influencing the organisation of the deprescribing process. It consists of two subthemes: (i) interprofessional collaboration and (ii) work processes, task division, and supporting roles.

#### Interprofessional collaboration

Community-dwelling patients with palliative needs were often under the shared care of various healthcare practitioners, including multiple prescribers.^38,40^ Healthcare practitioners described deprescribing as a collaborative endeavour, in which each professional discipline could make a distinct and valuable contribution based on their specific expertise and skill set. GPs were typically seen as holding primary responsibility for medication-related decisions.^40,41,50,53^ Regardless, accountability for deprescribing was occasionally perceived unclear by both healthcare practitioners and patients.^38,40,45,53,57^ Collaboration around the deprescribing process was often described by healthcare practitioners to be insufficient, challenging, and at times frustrating across all healthcare settings.^38,41,50,55,57^ Healthcare practitioners reported reluctance to alter colleagues’ prescriptions,^3,41,45,50,53,57^ though not all GPs shared this hesitation.^57^ Healthcare practitioners considered it ideal for relevant care providers, especially the prescribing physician, to be aligned on deprescribing decisions.^57^ Health care assistants occasionally felt disregarded when attempting to discuss medication-related concerns with physicians, GPs in particular, discouraging further input.^40^ Some GPs actively sought support from colleagues or experts in the deprescribing process, while others preferred making decisions independently.^3,49^

#### Work processes, task division, and supporting roles

Healthcare practitioners reported absence of systematic work processes for deprescribing in the palliative care context,^41,50^ including limited integration of deprescribing tools.^41,45^ Across all participants, there was consensus that implementing a structured, routine approach, including a clear delineation of tasks, could facilitate the deprescribing process.^41,45,50,53,58^ Physicians acknowledged that expectations regarding each discipline’s role differed, owing to the distinct competencies of professionals.^40,41,50^

Health care assistants perceived identifying potentially inappropriate medications as their primary role in the deprescribing process. Physicians supported this role, noting that deprescribing decisions could be informed by their observations. Nonetheless, nurses and GPs felt that medication-related recommendations proposed by health care assistants were often insufficiently substantiated.^40^

Pharmacist involvement in the deprescribing process was supported by all participants, particularly through medication reviews.^38,41^ Pharmacists were viewed as valuable sources of knowledge and support, and promoters of communication within healthcare teams.^38,45,57^ Some healthcare practitioners reported that pharmacists occasionally lacked insight into clinical complexity of patients with palliative needs and their estimated life expectancy.^40,50^

Participants agreed that nurses could contribute to the deprescribing process in patients with palliative needs.^40,48^ Physicians expressed differing views regarding the scope of their role, some noting it should be contingent upon their level of education.^48^ Nurses were described as having a signalling role through evaluation of the clinical situation, medication indications, and impact of medications on quality of life.^40,41,48,58^ The scope of their role further encompassed communicating observations and medication-related recommendations to physicians, while voicing patients’ and caregivers’ wishes, and informing and supporting patients and caregivers. The latter included assessment of caregivers’ capacity and aligning tasks accordingly.^40,41,45,48,58^

### Theme 4: The context of care

This theme entails prerequisites at a health system level and comprises one subtheme: (i) system-level factors.

#### System-level factors

Healthcare practitioners perceived time constraints to limit engagement in deprescribing and conducting peer consultations,^41,45,50,57^ which GPs partly attributed to the brevity of patient encounters in primary care and occasional challenges related to certain patients’ infrequent visits or poor adherence to follow-up.^57^ Continuity in monitoring the deprescribing process was complicated by the presence of many part-time and locum GPs and nurses.^50^ Enhancing workforce capacity and access to experts could help mitigate these barriers.^38,41,45,57^

A prescribing-oriented culture among physicians was described, consistent with guidelines’ primary focus on treatment initiation.^40,45,50,53^ System-level enablers of deprescribing included availability of adequate information and communications technology (ICT) facilities, with up-to-date and accessible electronic medical records that are interoperable across healthcare settings.^41,45,57^

## Discussion

### Main findings

This qualitative systematic review identified 23 publications examining factors influencing deprescribing in community-dwelling patients with palliative needs in primary care. Healthcare practitioners often perceived deprescribing as risky, due to clinical uncertainties, insufficient knowledge and training, and a lack of (awareness of) evidence-based guidance. They considered the process time-consuming, which, combined with time constraints and a high-risk perception, led them to assign it lower priority. Deprescribing conversations involved complex individualised evaluations of potential benefits and harms, and were ideally grounded in shared decision-making, incorporating holistic patient and caregiver assessments, and tailored communication and practical deprescribing strategies. When initiated by patients or caregivers, deprescribing was more likely to occur. Organisational facilitators included structured work processes, clear task delineation, and interprofessional collaboration. A prevailing culture favouring treatment initiation hindered deprescribing at the system level.

### What this study adds

This review demonstrates that primary healthcare practitioners have limited awareness of how to optimally enact deprescribing in community-dwelling patients with palliative needs, and continue to assign it a low priority, related to perceived risks, complexity, and time demands. Many healthcare practitioners were hesitant to initiate deprescribing conversations due to concerns about negative patient responses, despite evidence indicating that 92% of older adults are willing to consider deprescribing if recommended by their healthcare practitioner.^59^ This hesitancy appeared even more pronounced in prior reviews involving older adults in primary care.^15,19,20^ As treatment goals shift from curative intent to symptom management in palliative settings,^2^ deprescribing, particularly of preventive medications, may be clinically appropriate. Nonetheless, our findings suggest a greater complexity of individual considerations than reported in previous reviews in palliative care,^16–18^ which largely included studies conducted in residential care. Prognostic uncertainty may be higher in community settings, complicating benefit-harm assessments and timing of deprescribing. While certain tools can assist in prognostic estimation,^60,61^ some degree of uncertainty is unavoidable. Healthcare practitioners generally perceived continuing medications as safer than discontinuation, suggesting they underestimate potential harms of continued medication use and overestimate deprescribing risks. This heightened risk perception may be exacerbated by a paucity of intervention studies and limited awareness of existing deprescribing guidelines for populations with palliative needs,^62–64^ underscoring the need to strengthen, consolidate, and disseminate the deprescribing evidence base.^65^

Educational interventions for healthcare practitioners, focusing on deprescribing evidence, the use of available deprescribing tools and guidelines, and communication skills, may help reduce negative perceptions, promote awareness, and support making deprescribing a higher priority. Application of the Screening Tool of Older Persons Prescriptions in Frail adults with limited life expectancy (STOPPFrail), a validated instrument to assist with deprescribing in frail older adults with poor survival prognosis,^5,62^ was found to require only 2.7 min per patient after familiarisation,^66^ suggesting perceived time burden may be overstated. Educational interventions have previously demonstrated to improve medication appropriateness, either alone or within multifaceted approaches,^67–69^ and have been recommended to support physician engagement in deprescribing.^70^ Inadequate training has also been cited in deprescribing implementation studies as a barrier to successful implementation.^71,72^

This review further highlights the need for individualised, patient-centred deprescribing conversations, underpinned by shared decision-making, as noted in prior studies.^73–75^ Both patients and caregivers should ideally be involved in these conversations. Prior reviews in palliative care, predominantly based on studies in residential care,^16–18^ placed comparatively less emphasis on shared decision-making and patient-centredness than our findings and reviews involving older adults within primary care.^15,19,20^ As prognosis is often uncertain and deprescribing evidence remains limited, shared decision-making could be considered an ethical imperative. Nevertheless, findings from deprescribing implementation studies across palliative and other care contexts indicate that healthcare practitioners often accede to patient preferences when making deprescribing decisions.^72,76–78^ Whilst eliciting preferences is an essential component of meaningful deprescribing discussions, it should not deter healthcare practitioners from exploring individual barriers to medication cessation, which can be addressed accordingly through personalised communication. Geijteman et al.^79^ illustrated this through a case example demonstrating that addressing patient fears and framing deprescribing as a positive intervention can improve patient acceptance. Additionally, educating and empowering patients and caregivers fosters engagement and shared responsibility in deprescribing. This aligns with evidence showing that such approaches can effectively reduce medication use in outpatient settings.^80–83^

Strengthening interprofessional collaboration within individual practices and across healthcare settings is another important enabler. Baumgartner et al.^84^ identified interprofessional dynamics, particularly the relationship between pharmacists and the broader medical team, as the most commonly discussed determinant influencing implementation of deprescribing interventions in the elderly. Within each practice, healthcare practitioners should establish a clear task delineation, tailored to local contexts in terms of healthcare practitioners availability, time resources, trusting relationships, and competencies. Pharmacists are ideally positioned to contribute to the deprescribing process, a role widely recognised in primary care for its positive impact.^85,86^ The need for establishing systematic, routine work processes has been less emphasised in previous reviews in palliative care, possibly because transitions into residential care and close patient monitoring within these facilities naturally create more deprescribing opportunities and awareness. To ensure sustainability, deprescribing interventions should ideally be integrated within existing workflows, consistent with recommendations from prior deprescribing implementation research.^72,78,87^ With an increasing focus on advance care planning in primary care settings,^88^ it has been proposed to integrate deprescribing into advance care planning conversations,^89^ given their shared focus on aligning treatment with patient goals.^2^ Embedding deprescribing within advance care planning aligns with the broader cultural shift needed to support appropriate medication use for patients with palliative needs, from a prescribing-focused paradigm towards embedding routine medication assessments and deprescribing as standard practice. Realising such change requires education and training in deprescribing at both undergraduate and postgraduate levels, coupled with stronger inclusion of deprescribing principles within clinical guidelines.

Appropriate prescribing for patients with palliative needs is vital to improving palliative care quality. Medication appropriateness has recently been enshrined in the national quality indicators for palliative care in the Netherlands.^90^ This review provides guidance for clinical practice and the development of deprescribing implementation studies, which remain scarce.^91^ Overall, deprescribing in community-dwelling patients with palliative needs in primary care is precarious, yet it can be substantially improved by approaching it as a proactive, individualised, collaborative, and systematic practice.

### Strengths and limitations

This study was guided by a rigorous search strategy based on the PALETTE framework. Screening, data extraction, quality assessment, and coding were performed independently by two researchers, and we followed a team-based approach to discuss uncertainties and reach consensus on final interpretations. This improved the confirmability of our findings by reducing individual biases in data interpretation. Factors reported in lower quality studies were not unique and also appeared in studies deemed of appropriate quality.

All included studies were conducted in high-income Western countries, limiting the transferability of the findings to low- and middle-income countries. Moreover, restricting the search to publications in English and Dutch may limit transferability to other cultural contexts. Exclusion of grey literature may have increased the risk of publication bias. Many studies presented results combining settings and patient cohorts. In some cases, it was not possible to determine whether excerpts fully aligned with our inclusion criteria. In these instances, we opted to retain the sections in the analysis to preserve potentially relevant perspectives, which, despite conducting a sensitivity analysis, may have resulted in the inclusion of individual factors that are less relevant to our target population. Reflecting our exploratory aims, this review deliberately included studies conducted across diverse contexts, encompassing variation in primary care settings, countries, illness trajectories, and types of healthcare practitioners involved. As (de)prescribing practices may differ substantially across such contexts, individual factors are not expected to apply uniformly and should therefore be interpreted with attention to the local clinical and cultural conditions, which may shape their relevance and urgency. Although we highlighted context-specific nuances where feasible, contextual details could often not be disentangled or were insufficiently reported in the primary studies. Future research could further explore how contextual variation influences these factors.

## Conclusion

Deprescribing in community-dwelling patients with palliative needs in primary care can be optimised by employing a multidimensional strategy that includes patient-centredness, shared decision-making, education, collaboration within the multidisciplinary care team, and systematic work processes. Beyond individual consultations, a broader cultural shift is needed, from a prescribing-focused paradigm towards embedding deprescribing as a standard practice. Future research should prioritise integrating implementation science and expanding the evidence base for deprescribing, ultimately aiming to improve quality of life.

## Supplemental Material

sj-docx-1-pmj-10.1177_02692163261465769 – Supplemental material for Factors influencing deprescribing in community-dwelling patients with palliative needs in primary care: A qualitative systematic review Supplemental material, sj-docx-1-pmj-10.1177_02692163261465769 for Factors influencing deprescribing in community-dwelling patients with palliative needs in primary care: A qualitative systematic review by Jesse F. van Weelderen, Ankie C. M. Hazen, Alexa M. M. Mulder, Eric C. T. Geijteman, Saskia C. C. M. Teunissen, Dorien L. M. Zwart and Matthew P. Grant in Palliative Medicine
