# Supplementary material for: Factors influencing deprescribing in community-dwelling patients with palliative needs in primary care: A qualitative systematic review
Source: Palliat Med. 2026 Jul 31;40(9):1270–88. doi: 10.1177/02692163261465769 (PMC13612883; doi:10.1177/02692163261465769)
Supplement: Supplementary file 1 [file sj-docx-1-pmj-10.1177_02692163261465769.docx]

**Supplemental Table 1.** Search strategy MEDLINE.
"Deprescriptions"[MeSH Terms] OR "Withholding Treatment"[MeSH Terms] OR "Inappropriate Prescribing"[MeSH Terms] OR "Potentially Inappropriate Medication List"[MeSH Terms] OR "Medical Futility"[MeSH Terms] OR "deprescri*"[Title/Abstract] OR "discontinu*"[Title/Abstract] OR "taper*"[Title/Abstract] OR (("reduc*"[Title/Abstract] OR "stop*"[Title/Abstract] OR "withdraw*"[Title/Abstract] OR "cessation"[Title/Abstract] OR "ceas*"[Title/Abstract] OR "rationali*"[Title/Abstract] OR "reconcil*"[Title/Abstract] OR "withhold*"[Title/Abstract] OR "diminish*"[Title/Abstract] OR "deintensif*"[Title/Abstract] OR "inappropriate"[Title/Abstract] OR "unnecessary"[Title/Abstract] OR "non-essential"[Title/Abstract] OR "preventive"[Title/Abstract] OR "limited benefit"[Title/Abstract] OR "limited usefulness"[Title/Abstract] OR "questionable benefit"[Title/Abstract] OR "questionable usefulness"[Title/Abstract]) AND ("medication*"[Title/Abstract] OR "medicine*"[Title/Abstract] OR "drug*"[Title/Abstract] OR "prescri*"[Title/Abstract])) OR "drug* review*"[Title/Abstract] OR "dose reduction*"[Title/Abstract] OR "medication* management" [Title/Abstract] OR "medic* review*"[Title/Abstract]

AND

("Primary care"[Title/Abstract] OR "primary health care"[Title/Abstract] OR "Primary healthcare"[Title/Abstract] OR "general pract*"[Title/Abstract] OR "communit*"[Title/Abstract] OR "ambulator*"[Title/Abstract] OR "Family"[Title/Abstract] OR "GP"[Title/Abstract] OR "GPs"[Title/Abstract] OR "healthcare professional"[Title/Abstract] OR "health care professional"[Title/Abstract] OR "primary health care"[MeSH Terms] OR "Family Practice"[MeSH Terms] OR "physicians, family"[MeSH Terms] OR "General Practitioners"[MeSH Terms] OR "General Practice"[MeSH Terms])

AND

("palliat*"[Title/Abstract] OR "life limiting illness*"[Title/Abstract] OR "life threatening illness*"[Title/Abstract] OR "serious illness*"[Title/Abstract] OR "advanced illness*"[Title/Abstract] OR "incurable illness*"[Title/Abstract] OR "life limiting condition*"[Title/Abstract] OR "life threatening condition*"[Title/Abstract] OR "serious condition*"[Title/Abstract] OR "advanced condition*"[Title/Abstract] OR "incurable condition*"[Title/Abstract] OR "life limiting disease*"[Title/Abstract] OR "life threatening disease*"[Title/Abstract] OR "serious disease*"[Title/Abstract] OR "advanced disease*"[Title/Abstract] OR "incurable disease*"[Title/Abstract] OR "limited life expectancy"[Title/Abstract] OR "diminished life expectancy"[Title/Abstract] OR "short life expectancy"[Title/Abstract] OR "end of life"[Title/Abstract] OR "Eol"[Title/Abstract] OR "terminal*"[Title/Abstract] OR "Poor prognosis"[Title/Abstract] OR "Poor survival prognosis"[Title/Abstract] OR "frail*"[Title/Abstract] OR "advanced cancer"[Title/Abstract] OR "multimorbid*"[Title/Abstract] OR "Terminal Care"[MeSH Terms] OR "Palliative Care"[MeSH Terms] OR "Palliative Medicine"[Mesh Terms] OR "Terminally Ill"[MeSH Terms] OR "Frail Elderly"[MeSH Terms] OR "Frailty"[MeSH Terms])

AND

("interview*"[Title/Abstract] OR "qualitative"[Title/Abstract] OR "mixed method*"[Title/Abstract] OR "multimethod*"[Title/Abstract] OR "phenomenolog*"[Title/Abstract] OR (("semi structured"[Title/Abstract] OR "semistructured"[Title/Abstract] OR "unstructured"[Title/Abstract] OR "structured"[Title/Abstract] OR "indepth"[Title/Abstract] OR "in depth"[Title/Abstract] OR "face-to-face"[Title/Abstract] OR "informal"[Title/Abstract] OR "guid*"[Title/Abstract]) AND ("interview*"[Title/Abstract] OR "discussion*"[Title/Abstract])) OR "focus group*"[Title/Abstract] OR "focusgroup*"[Title/Abstract] OR "ethnograph*"[Title/Abstract] OR "fieldwork"[Title/Abstract] OR "Field work"[Title/Abstract] OR "narrati*"[Title/Abstract] OR "key informant"[Title/Abstract] OR "questionnaire*"[Title/Abstract] OR "survey*"[Title/Abstract] OR "qualitative research"[MeSH Terms] OR "interviews as topic"[MeSH Terms] OR "narration"[MeSH Terms] OR "personal narratives as topic"[MeSH Terms] OR "focus groups"[MeSH Terms])

Filters: English, Dutch, 2000 – January 2025.

**Supplemental Table 2 and 3.** Quality assessment.

| **Supplemental Table 2.** Critical appraisal of the included qualitative studies according to the CASP Checklist for Qualitative Research.^1^ ^a^ | | | | | | | | | | | |
| --- | --- | --- | --- | --- | --- | --- | --- | --- | --- | --- | --- |
| Study details (first author, publication year) | Clear aims | Appropriate qualitative methodology | Appropriate research design | Appropriate recruitment strategy | Appropriate data collection | Consideration Researcher-participant relationship | Consideration ethical issues | Rigorous data analysis | Clear findings | Valuable research | Total score^b^ |
| **Alwidyan (2023)^2^** | Y | Y | Y | Y | Y | Y | Y | Y | Y | Y | 10 |
| **Cortis (2017)^3^** | Y | N | U | Y | U | U | Y | U | N | Y | 4 |
| **Dees (2018)^4^** | Y | Y | U | Y | Y | U | Y | Y | Y | Y | 8 |
| **Geijteman (2016)^5^** | Y | Y | U | Y | Y | U | U | U | Y | Y | 6 |
| **Hall (2023)^6^** | Y | Y | U | Y | Y | Y | Y | Y | Y | Y | 9 |
| **Huisman (2020)^7^** | Y | Y | U | Y | U | U | Y | Y | Y | Y | 7 |
| **Kuruvilla (2018)^8^** | Y | Y | U | U | Y | Y | Y | U | Y | Y | 7 |
| **Lundby (2019)^9^** | Y | Y | Y | Y | Y | U | Y | Y | Y | Y | 9 |
| **McCloskey (2018)^10^** | Y | Y | U | U | Y | Y | Y | U | Y | Y | 7 |
| **Mc Namara (2017)^11^** | Y | Y | U | Y | Y | Y | Y | Y | Y | Y | 9 |
| **Parsons (2019)^12^** | Y | Y | Y | U | Y | Y | Y | Y | Y | N | 8 |
| **Robinson-Barrella (2014)^13^** | Y | Y | U | Y | Y | Y | Y | Y | Y | Y | 9 |
| **Sand (2018)^14^** | Y | Y | U | Y | Y | U | Y | Y | Y | Y | 8 |
| **Sheard (2012)^15^** | Y | Y | U | Y | U | U | Y | Y | N | N | 5 |
| **Tjia (2020)^16^** | Y | Y | U | U | U | U | U | Y | Y | Y | 5 |
| **Todd (2016)^17^** | Y | Y | Y | Y | Y | U | Y | Y | Y | Y | 9 |
| **Van der Waal (2024)^18^** | Y | Y | Y | Y | Y | Y | Y | Y | Y | Y | 10 |
| **Van Middelaar (2018)^19^** | Y | Y | U | Y | Y | Y | Y | Y | Y | Y | 9 |
| **Wyatt (2022)^20^** | Y | Y | U | Y | Y | U | Y | Y | Y | Y | 8 |
| Y = Yes, meets criterium, N = No, doesn’t meet criterium, U = Unclear if meets criterium. ^a^CASP: Critical Appraisal Skills Programme: Checklist for Qualitative Research.  ^b^Total quality assessment score on a scale from 0-10 based on the number of criteria being met. | | | | | | | | | | | |

| Supplemental Table 3. Critical appraisal of the included mixed-methods studies according to the Mixed Methods Appraisal Tool (MMAT).^21^ | | | | |
| --- | --- | --- | --- | --- |
|  | **Huisman (2021)^22^** | **Huisman (2021)^23^** | **Parsons (2014)^24^** | **Pype (2018)^25^** |
| Clear research questions | Y | Y | Y | Y |
| Data allow to address research questions | Y | Y | Y | Y |
| Appropriate qualitative approach | Y | Y | U | Y |
| Adequate qualitative data collection methods | Y | N | N | Y |
| Qualitative findings adequately derived from data | Y | N | Y | U |
| Interpretation qualitative results sufficiently substantiated | Y | N | N | Y |
| Coherence between qualitative data sources, collection, analysis and interpretation | Y | N | N | Y |
| Relevant sampling strategy | Y | N | Y | N |
| Representative sample of target population | U | N | U | N |
| Appropriate measurements | Y | Y | Y | Y |
| Low risk nonresponse bias | N | Y | N | Y |
| Appropriate statistical analysis | Y | Y | Y | Y |
| Adequate rationale mixed methods design | U | U | U | Y |
| Effective integration different components | Y | N | N | Y |
| Outputs of integration of qualitative and quantitative components adequately interpreted | Y | N | N | Y |
| Divergences and inconsistencies between quantitative and qualitative results adequately addressed | U | N | N | U |
| Different components adhere to the quality criteria of each tradition of involved methods | Y | N | N | Y |
| Total score^a^ | 7.6 | 3.5 | 3.5 | 7.6 |
| Y = Yes, meets criterium, N = No, doesn’t meet criterium, U = Unclear if meets criterium. ^a^Total quality assessment score on a scale from 0-10 based on the number of criteria being met/17 x 10. | | | | |

**Supplemental Table 4.** List of excluded articles following full-text screening and their reason of exclusion.

| **Reference** | **Reason for exclusion** |
| --- | --- |
| Abbott LJ. PROACTIVE FRAILTY MANAGEMENT WITHIN AN INTEGRATED CARE SYSTEM. InAGE AND AGEING 2019 Feb 1 (Vol. 48). GREAT CLARENDON ST, OXFORD OX2 6DP, ENGLAND: OXFORD UNIV PRESS. | Wrong publication type |
| Abuzour AS, Wilson SA, Woodall AA, Mair FS, Clegg A, Shantsila E, Gabbay M, Abaho M, Aslam A, Bollegala D, Cant H. A qualitative exploration of barriers to efficient and effective structured medication reviews in primary care: findings from the DynAIRx study. Plos one. 2024 Aug 30;19(8):e0299770. | Wrong population |
| Aharaz A, Rasmussen JH, McNulty HB, Cyron A, Fabricius PK, Bengaard AK, Sejberg HR, Simonsen RR, Treldal C, Houlind MB. A collaborative deprescribing intervention in a subacute medical outpatient clinic: A pilot randomized controlled trial. Metabolites. 2021 Mar 30;11(4):204. | Wrong population |
| Ailabouni NJ, Nishtala PS, Mangin D, Tordoff JM. General practitioners’ insight into deprescribing for the multimorbid older individual: a qualitative study. International Journal of Clinical Practice. 2016 Mar;70(3):261-76. | Wrong setting |
| Akyon SH, Akyon FC, Yılmaz TE. Artificial intelligence-supported web application design and development for reducing polypharmacy side effects and supporting rational drug use in geriatric patients. Frontiers in Medicine. 2023 Mar 8;10:1029198. | Wrong population |
| Ali, Abid & Latif, Asam. (2020). Patient and lay family care giver perspectives on community palliative care and the community pharmacist involvement. | Wrong publication type |
| Ali S, Curtain CM, Peterson GM, Salahudeen MS. Exploring Australian pharmacists' views toward reducing the risk of medicines-related harm in aged care residents. Pharmacol Res Perspect. 2023 Jun;11(3):e01104. doi: 10.1002/prp2.1104. PMID: 37222482; PMCID: PMC10207935. | Wrong setting |
| D Allcock, E Page, S McCracken, E Thorman, R Marchant, C Worth, H Fraser, D Shipway, 1603 PROACTIVE ADVANCE CARE PLANNING ALONGSIDE COMPREHENSIVE GERIATRIC ASSESSMENT IN CARE HOMES: A QUALITATIVE FEEDBACK ANALYSIS, *Age and Ageing*, Volume 52, Issue Supplement_2, July 2023, afad104.098, <https://doi.org/10.1093/ageing/afad104.098> | Wrong publication type |
| Alves A, Green S, James DH. Deprescribing of Medicines in Care Homes-A Five-Year Evaluation of Primary Care Pharmacist Practices. Pharmacy (Basel). 2019 Aug 3;7(3):105. doi: 10.3390/pharmacy7030105. PMID: 31382582; PMCID: PMC6789793. | Wrong publication type |
| Anderson K, Foster M, Freeman C, Luetsch K, Scott I. Negotiating "Unmeasurable Harm and Benefit": Perspectives of General Practitioners and Consultant Pharmacists on Deprescribing in the Primary Care Setting. Qual Health Res. 2017 Nov;27(13):1936-1947. doi: 10.1177/1049732316687732. Epub 2017 Jan 12. PMID: 29088989. | Wrong population |
| Armitage S. STOPP (Screening Tool of Older Person's Prescriptions) and START (Screening Tool to Alert doctors to Right Treatment) as a pharmacy service  *European Journal of Hospital Pharmacy* 2012;19:221. | Wrong publication type |
| Armstrong I, Yorke J, Harries C, Bundock S, Rochia N. Pulmonary Arterial Hypertension: A Qualitative Investigation Of Patients Views Regarding Treatments. InC63. MANAGEMENT OF PULMONARY HYPERTENSION 2012 May (pp. A4810-A4810). American Thoracic Society. | Wrong publication type |
| Babakhanians, A., Gurvich, T., Sehgal, S. Addressing Polypharmacy in Geriatric Patient Population through an Interdisciplinary Care Approach: A Case Report. J. Am. Geriatr. Soc. - Volume 71, Issue 0, pp. S259-S260 - published 2023-01-01 | Wrong publication type |
| Bacon M, Kematick B, Suliman I, Scullion B, Chua I, Given S, Lally K. Development of PharmPAL: A Collaborative Practice Pharmacy Clinic in Ambulatory Palliative Care. Am J Hosp Palliat Care. 2023 May;40(5):475-479. doi: 10.1177/10499091221120593. Epub 2022 Sep 2. PMID: 36052826. | Wrong phenomena of interest |
| Balwan, S., Jervis, R., Conigliaro, J., Smith, K.L. Bridging the chasm-advanced illness management: Higher quality, lower cost. J. Gen. Intern. Med. - Volume 29, Issue 0, pp. S479 - published 2014-01-01 | Wrong publication type |
| Barkhausen T, Junius-Walker U, Hummers-Pradier E, Mueller CA, Theile G. "It's MAGIC"--development of a manageable geriatric assessment for general practice use. BMC Fam Pract. 2015 Jan 22;16:4. doi: 10.1186/s12875-014-0215-4. PMID: 25608946; PMCID: PMC4320637. | Wrong phenomena of interest |
| Barnato AE, Anthony DL, Skinner J, Gallagher PM, Fisher ES. Racial and ethnic differences in preferences for end-of-life treatment. J Gen Intern Med. 2009 Jun;24(6):695-701. doi: 10.1007/s11606-009-0952-6. Epub 2009 Apr 23. PMID: 19387750; PMCID: PMC2686762. | Wrong phenomena of interest |
| Baughman, A.W., Murphy, N., Driscoll, L et al. Improving medication reconciliation in a veterans affairs skilled nursing facility as part of MARQUIS2. J. Gen. Intern. Med. - Volume 33, Issue 2, pp. 804-805 - published 2018-01-01 | Wrong publication type |
| Beccaro M, Lora Aprile P, Scaccabarozzi G, Cancian M, Costantini M. Survey of Italian general practitioners: knowledge, opinions, and activities of palliative care. J Pain Symptom Manage. 2013 Sep;46(3):335-44. doi: 10.1016/j.jpainsymman.2012.08.020. Epub 2012 Nov 27. PMID: 23195391. | Wrong phenomena of interest |
| Bell HT, Granas AG, Enmarker I, Omli R, Steinsbekk A. Nurses' and pharmacists' learning experiences from participating in interprofessional medication reviews for elderly in primary health care - a qualitative study. BMC Fam Pract. 2017 Feb 28;18(1):30. doi: 10.1186/s12875-017-0598-0. PMID: 28241789; PMCID: PMC5330158. | Wrong population |
| Bennett, J. Managing problematic polypharmacy: GP trainee perspectives on their role in deprescribing. Br. J. Gen. Pract. - Volume 70, Issue 0, pp. - published 2020-01-01 | Wrong publication type |
| Bennett MI, Allsop MJ, Allen P, Allmark C, Bewick BM, Black K, Blenkinsopp A, Brown J, Closs SJ, Edwards Z, Flemming K, Fletcher M, Foy R, Godfrey M, Hackett J, Hall G, Hartley S, Howdon D, Hughes N, Hulme C, Jones R, Meads D, Mulvey MR, O’Dwyer J, Pavitt SH, Rainey P, Robinson D, Taylor S, Wray A, Wright-Hughes A, Ziegler L. Pain self-management interventions for community-based patients with advanced cancer: a research programme including the IMPACCT RCT. Southampton (UK): NIHR Journals Library; 2021 Dec. PMID: 34870925. | Wrong phenomena of interest |
| Bertozzo, G. & Granziera, Serena & Petruzzellis, F. & Marigo, L. & Rossi, K. & Infante, T. & Pletti, S. & Nante, Giovanni & Manzato, Enzo. (2013). Causes for vitamin K antagonist discontinuation in elderly patients with atrial fibrillation. European Geriatric Medicine. 4. S42-S43. 10.1016/j.eurger.2013.07.140. | Wrong publication type |
| Birt, L., Wright, D. J., Blacklock, J., Bond, C. M., Hughes, C. M., Alldred, D. P., Holland, R., & Scott, S. (2022). Enhancing deprescribing: A qualitative understanding of the complexities of pharmacist-led deprescribing in care homes. Health & Social Care in the Community, 30, e6521–e6531. <https://doi.org/10.1111/hsc.14099> | Wrong setting |
| Bolmsjö BB, Palagyi A, Keay L, Potter J, Lindley RI. Factors influencing deprescribing for residents in Advanced Care Facilities: insights from General Practitioners in Australia and Sweden. BMC Fam Pract. 2016 Nov 5;17(1):152. doi: 10.1186/s12875-016-0551-7. PMID: 27814691; PMCID: PMC5097406. | Wrong publication type |
| Bosshard G, Nilstun T, Bilsen J, Norup M, Miccinesi G, van Delden JJ, Faisst K, van der Heide A; European End-of-Life Consortium. Forgoing treatment at the end of life in 6 European countries. Arch Intern Med. 2005 Feb 28;165(4):401-7. doi: 10.1001/archinte.165.4.401. PMID: 15738368 | Wrong phenomena of interest |
| Bradburn, L., McNair, S., Munang, L.A. STRUCTURED MULTIDISCIPLINARY REVIEWS FOR CARE HOME RESIDENTS REDUCE POLYPHARMACY COST-EFFECTIVELY. Age Ageing - Volume 52, Issue 0, pp. ii6 - published 2023-01-01 | Wrong publication type |
| Braun UK, Beyth RJ, Ford ME, McCullough LB. Voices of African American, Caucasian, and Hispanic surrogates on the burdens of end-of-life decision making. J Gen Intern Med. 2008 Mar;23(3):267-74. doi: 10.1007/s11606-007-0487-7. Epub 2008 Jan 3. PMID: 18172738; PMCID: PMC2359470. | Wrong phenomena of interest |
| L Breen, J Benn, I Marques, G Peat, B Fylan, J Silcock, D Mehdizadeh, D Petty, A Daffu-O’Reilly, N Butt, D P Alldred, 482 Developing a person-centred deprescribing process in primary care for older people living with frailty, *International Journal of Pharmacy Practice*, Volume 31, Issue Supplement_1, April 2023, Page i27, <https://doi.org/10.1093/ijpp/riad021.031> | Wrong publication type |
| Brooks CF, Argyropoulos A, Matheson-Monnet CB, Kryl D. Evaluating the impact of a polypharmacy Action Learning Sets tool on healthcare practitioners' confidence, perceptions and experiences of stopping inappropriate medicines. BMC Med Educ. 2022 Jun 27;22(1):499. doi: 10.1186/s12909-022-03556-8. PMID: 35761284; PMCID: PMC9235240. | Wrong population |
| Brown A, Wills M, Francis S*, et al.* Patient safety consequences of medication incidents in palliative care: mixed method analysis of incident reports. *BMJ Supportive & Palliative Care* 2022;12:A17. | Wrong publication type |
| Brown, T., Link, R. Symptom management in a palliative care program. J. Palliative Med. - Volume 21, Issue 5, pp. A24 - published 2018-01-01 | Wrong publication type |
| Cadogan, C. A., Ryan, C., Gormley, G. J., Passmore, P., Francis, J., Kerse, N., & Hughes, C. (2015). Prescribing appropriate polypharmacy for older people in primary care: a qualitative study of general practitioners’ perceptions and experiences. International Journal of Pharmacy Practice, 23(S1), 9-10. <https://doi.org/10.1111/ijpp.12186> | Wrong publication type |
| Cadogan, Cathal & Ryan, Cristin & Gormley, GJ & Passmore, P & Francis, Jill & Kerse, Ngaire & Hughes, Carmel. (2015). Dispensing appropriate polypharmacy to older people in primary care: a qualitative, theory-based study of community pharmacists’ perceptions and experiences.. | Wrong publication type |
| Campbell C, Ramakuela J, Dennis D, Soba K. Palliative Care Practices in a Rural Community: Cultural Context and the Role of Community Health Worker. J Health Care Poor Underserved. 2021;32(1):550-564. doi: 10.1353/hpu.2021.0040. PMID: 33678713. | Wrong phenomena of interest |
| Carrier H, Zaytseva A, Bocquier A, Villani P, Verdoux H, Fortin M, Verger P. GPs' management of polypharmacy and therapeutic dilemma in patients with multimorbidity: a cross-sectional survey of GPs in France. Br J Gen Pract. 2019 Apr;69(681):e270-e278. doi: 10.3399/bjgp19X701801. Epub 2019 Feb 25. PMID: 30803978; PMCID: PMC6428490. | Wrong population |
| Chadborn NH, Devi R, Williams C, Sartain K, Goodman C, Gordon AL. GPs’ involvement to improve care quality in care homes in the UK: a realist review. Southampton (UK): NIHR Journals Library; 2021 Oct. PMID: 34730923. | Wrong publication type |
| Coelho T, Rosendo I, Seiça Cardoso C. Evaluation of deprescription by general practitioners in elderly people with different levels of dependence: cross-sectional study. BMC Prim Care. 2024 Mar 2;25(1):78. doi: 10.1186/s12875-024-02299-3. PMID: 38431577; PMCID: PMC10908147. | Wrong population |
| Downing NJ, Skaczkowski G, Hughes-Barton D, Stone H, Robinson L, Gunn KM. A qualitative exploration of the role of a palliative care pharmacist providing home-based care in the rural setting, from the perspective of health care professionals. Aust J Rural Health. 2024 Jun;32(3):510-520. doi: 10.1111/ajr.13115. Epub 2024 Mar 27. PMID: 38544325. | Wrong phenomena of interest |
| Drewelow E, Ritzke M, Altiner A, Icks A, Montalbo J, Kalitzkus V, Löscher S, Pashutina Y, Fleischer S, Abraham J, Thürmann P, Mann NK, Wiese B, Wilm S, Wollny A, Feldmeier G, Buuck T, Mortsiefer A; COFRAIL study group. Development of a shared decision-making intervention to improve drug safety and to reduce polypharmacy in frail elderly patients living at home. PEC Innov. 2022 Mar 24;1:100032. doi: 10.1016/j.pecinn.2022.100032. PMID: 37213749; PMCID: PMC10194292. | Wrong population |
| Driller, B., Karlsen, L., Winsjansen, B., Strømskag, K.E. Implementation of liverpool care pathway for the dying patient (LCP) in primary health care in Norway. Palliative Med. - Volume 28, Issue 6, pp. 776 - published 2014-01-01 | Wrong publication type |
| Durand M, Forestier E, Gras Aygon C, Sirvain S, de Wazières B, Bernard L, Paccalin M, Legout L, Roubaud Baudron C, Gavazzi G, Fraisse T; L’intergroupe GInGer (SPILF SFGG). Determinants of doctors' antibiotic prescriptions for patients over 75 years old in the terminal stage of palliative care. Infect Dis Now. 2021 Jun;51(4):340-345. doi: 10.1016/j.medmal.2020.10.013. Epub 2020 Oct 16. PMID: 33075403. | Wrong phenomena of interest |
| El-Dahiyat F, Jairoun AA, Al-Hemyari SS, Shahwan M, Hassan N, Jairoun S, Jaber AAS. Are pharmacists' knowledge and practice the key to promoting deprescribing of potentially inappropriate medication: a missing link between treatment and outcomes. Int J Pharm Pract. 2023 Jun 30;31(4):387-395. doi: 10.1093/ijpp/riad027. PMID: 37116892. | Wrong population |
| Elwyn G, Williams M, Roberts C, Newcombe RG, Vincent J. Case management by nurses in primary care: analysis of 73 'success stories'. Qual Prim Care. 2008;16(2):75-82. PMID: 18700083. | Wrong phenomena of interest |
| Emblin, K., Dilks, S., Nash, I., Fowler, S., Smith, G., Mulcock, K., Jefferies, S. Exeter cluster pharmacy service evaluation demonstrates hospital admission avoidance. Int. J. Pharm. Pract. - Volume 23, Issue 0, pp. 82 - published 2015-01-01 | Wrong publication type |
| Ethier JL, Paramsothy T, You JJ, Fowler R, Gandhi S. Perceived Barriers to Goals of Care Discussions With Patients With Advanced Cancer and Their Families in the Ambulatory Setting: A Multicenter Survey of Oncologists. J Palliat Care. 2018 Jul;33(3):125-142. doi: 10.1177/0825859718762287. Epub 2018 Apr 2. PMID: 29607704. | Wrong setting |
| Fenton ATHR, Ornstein KA, Kent EE, Miller-Sonet E, Wright AA, Dionne-Odom JN. A comparison of adult-child and spousal cancer caregivers' participation in medical decisions. PLoS One. 2024 Jun 13;19(6):e0300450. doi: 10.1371/journal.pone.0300450. PMID: 38870142; PMCID: PMC11175391. | Wrong setting |
| Foley L, Hynes L, Murphy AW, Molloy GJ. 'Just keep taking them, keep hoping they'll work': A qualitative study of adhering to medications for multimorbidity. Br J Health Psychol. 2022 Sep;27(3):691-715. doi: 10.1111/bjhp.12568. Epub 2021 Oct 31. PMID: 34719079. | Wrong population |
| Foley, Mary & Hickey, Mary & Timmons, Suzanne & Harnedy, Norma & Crowley, Maria & Agar, Martina. (2014). The Pivotal Role of the Assessment and Treatment Centre/Day Hospital in Optimizing Health, Function and Wellness in Older Adults. Irish Journal of Medical Science. 183. S340-S340. | Wrong publication type |
| Folwell et al. BRINGING COMPREHENSIVE GERIATRIC ASSESSMENT TO CARE HOMES - HULL PILOT, *Age and Ageing*, Volume 47, Issue suppl_2, April 2018, Pages ii25–ii39, <https://doi.org/10.1093/ageing/afy028.59> | Wrong publication type |
| S A Francis, J Elyan, M Ogden, B D Franklin, K L Mattick, A Kajamaa, S Yardley, Prescribing and medication use for symptom control in palliative care: stakeholder engagement within an activity theory study, *International Journal of Pharmacy Practice*, Volume 30, Issue Supplement_1, April 2022, Pages i43–i44, <https://doi.org/10.1093/ijpp/riac019.061> | Wrong publication type |
| Friedl, J., Badrane, L., Linck, S., Dory, A., Amé, S., Kurtz, J.E., Gourieux, B. Drug interactions in ambulatory cancer patients. Eur. J. Oncolog. Pharm. - Volume 1, Issue 3, pp. 38-39 - published 2018-01-01 | Wrong publication type |
| Gaël L, Nadine K, Sandra B, Rachida B. General practitioners' views on inappropriate prescribing for older patients: a qualitative study through focus groups. BMC Geriatr. 2024 Oct 18;24(1):847. doi: 10.1186/s12877-024-05451-x. PMID: 39425021; PMCID: PMC11492209. | Wrong population |
| Garcia, W.J., Genao, M., Speight, I., Ang, K., Brennan, M. Falls prevention model for geriatric/palliative house calls program. J. Am. Geriatr. Soc. - Volume 67, Issue 0, pp. S215 - published 2019-01-01 | Wrong publication type |
| Gaup, Mari-Ann & Halvorsen, Kjell. (2015). Physicians' experiences with NORGEP criteria and the use of inappropriate medication in elderly patients in nursing home and home care service. International Journal of Clinical Pharmacy. 37. 14-15. | Wrong publication type |
| Gaw CE, Hamilton KW, Gerber JS, Szymczak JE. Physician Perceptions Regarding Antimicrobial Use in End-of-Life Care. Infect Control Hosp Epidemiol. 2018 Apr;39(4):383-390. doi: 10.1017/ice.2018.6. Epub 2018 Feb 12. PMID: 29428002. | Wrong setting |
| Geijteman et al. Understanding continuation of potentially inappropriate medications at the end of life: Perspectives from patients, their relatives and physicians EAPC2016: Abstracts. *Palliative Medicine*. 2016;30(6):NP1-NP401. doi:[10.1177/0269216316646056](https://doi.org/10.1177/0269216316646056) | Wrong publication type |
| Geijteman ECT, Huisman BAA, Dees MK, Perez RSGM, van der Rijt CCD, van Zuylen L, van der Heide A. Medication Discontinuation at the End of Life: A Questionnaire Study on Physicians' Experiences and Opinions. J Palliat Med. 2018 Aug;21(8):1166-1170. doi: 10.1089/jpm.2017.0501. Epub 2018 Apr 18. PMID: 29668346. | Wrong phenomena of interest |
| Gerlach N, Michiels-Corsten M, Viniol A, Schleef T, Junius-Walker U, Krause O, Donner-Banzhoff N. Professional roles of general practitioners, community pharmacists and specialist providers in collaborative medication deprescribing - a qualitative study. BMC Fam Pract. 2020 Sep 4;21(1):183. doi: 10.1186/s12875-020-01255-1. PMID: 32887551; PMCID: PMC7487755. | Wrong population |
| Giannitrapani, Azarfar, Silveira, Midboe, Glassman, Becker, Lorenz, Karl et al. Shared Decision Making for Long-Term Opioid Therapy for Cancer Pain (SA528A). Journal of Pain and Symptom Management, Volume 57, Issue 2, 453 - 454 | Wrong publication type |
| Hand et al. Abstracts from the 2021 Annual Meeting of the Society of General Internal Medicine. J GEN INTERN MED 36 (Suppl 1), 1–469 (2021). https://doi.org/10.1007/s11606-021-06830-5 | Wrong publication type |
| Hansen L, Archbold PG, Stewart B, Westfall UB, Ganzini L. Family caregivers making life-sustaining treatment decisions: factors associated with role strain and ease. J Gerontol Nurs. 2005 Nov;31(11):28-35. doi: 10.3928/0098-9134-20051101-08. PMID: 16320432. | Wrong phenomena of interest |
| Harriman, Kasandra & Howard, Lisa & McCracken, Rita. (2014). Deprescribing medication for frail elderly patients in nursing homes: A survey of Vancouver family physicians.. British Columbia Medical Journal. 56. 436-441. | Wrong publication type |
| Haverhals LM, Lee CA, Siek KA, Darr CA, Linnebur SA, Ruscin JM, Ross SE. Older adults with multi-morbidity: medication management processes and design implications for personal health applications. J Med Internet Res. 2011 Jun 29;13(2):e44. doi: 10.2196/jmir.1813. PMID: 21715286; PMCID: PMC3221383. | Wrong population |
| Heckman GA, Gimbel S, Mensink C, Kroetsch B, Jones A, Nasim A, Northwood M, Elliott J, Morrison A. The Integrated Care Team: A primary care based-approach to support older adults with complex health needs. Healthc Manage Forum. 2024 Oct 22:8404704241293051. doi: 10.1177/08404704241293051. Epub ahead of print. PMID: 39434587 | Wrong population |
| Heinrich, C.H., McCarthy, S., McHugh, S., Curran, G.M., Donovan, M.D. Multidisciplinary deprescribing review for frail older adults in long-term care: Development of a theory informed implementation strategy using a Delphi survey and Roundtable discussion with stakeholders. Int. J. Pharm. Pract. - Volume 31, Issue 0, pp. i8-i9 - published 2023-01-01 | Wrong publication type |
| C H Heinrich, S McCarthy, S McHugh, E R Shanahan, M D Donovan, Feasibility study of a multidisciplinary DEprescribing review for Frail oldER Adults in Long-term care (DEFERAL) targeting potentially inappropriate antihypertensive medications, *International Journal of Pharmacy Practice*, Volume 32, Issue Supplement_1, April 2024, Pages i16–i17, <https://doi.org/10.1093/ijpp/riae013.021> | Wrong publication type |
| Heinrich CH, McHugh S, McCarthy S, Curran GM, Donovan MD. Multidisciplinary DEprescribing review for Frail oldER adults in long-term care (DEFERAL): Implementation strategy design using behaviour science tools and stakeholder engagement. Res Social Adm Pharm. 2023 Aug;19(8):1202-1213. doi: 10.1016/j.sapharm.2023.05.002. Epub 2023 May 12. PMID: 37230873. | Wrong setting |
| Hermsen M, ten Have H. Decision-making in palliative care practice and the need for moral deliberation: a qualitative study. Patient Educ Couns. 2005 Mar;56(3):268-75. doi: 10.1016/j.pec.2004.03.013. PMID: 15721968. | Wrong phenomena of interest |
| Hickman, S. E. , Weaver, M. T. , Habermann, B. & Helft, P. R. (2014). *Journal of Clinical Oncology, 32* (31), 101. doi: 10.1200/jco.2014.32.31_suppl.101. | Wrong publication type |
| Hinkka H, Kosunen E, Metsänoja R, Lammi UK, Kellokumpu-Lehtinen P. Factors affecting physicians' decisions to forgo life-sustaining treatments in terminal care. J Med Ethics. 2002 Apr;28(2):109-14. doi: 10.1136/jme.28.2.109. PMID: 11934941; PMCID: PMC1733551. | Wrong setting |
| K.P.G.M. Hurkens, C. Mestres-Gonzalvo, H.A.J.M. de Wit, P.H.M. van der Kuy, R. Janknegt, F. Verhey, J.M.G.A. Schols, C.D.A. Stehouwer, W. Mulder. Medication reviews in elderly patients: Substantial variation in daily practice, European Geriatric Medicine, Volume 4, Supplement 1,  2013, pS182-S183, ISSN 1878-7649, <https://doi.org/10.1016/j.eurger.2013.07.611> | Wrong publication type |
| Hussainy SY, Box M, Scholes S. Piloting the role of a pharmacist in a community palliative care multidisciplinary team: an Australian experience. BMC Palliat Care. 2011 Oct 31;10:16. doi: 10.1186/1472-684X-10-16. PMID: 22035160; PMCID: PMC3215169. | Wrong phenomena of interest |
| Ie K, Machino R, Albert SM, Tomita S, Kushibuchi M, Hirose M, Matsuda T, Okuse C, Ohira Y. Deprescribing as an Opportunity to Facilitate Patient-Centered Care: A Qualitative Study of General Practitioners and Pharmacists in Japan. Int J Environ Res Public Health. 2023 Feb 17;20(4):3543. doi: 10.3390/ijerph20043543. PMID: 36834238; PMCID: PMC9962748. | Wrong population |
| Inch J, Notman F, Bond CM, Alldred DP, Arthur A, Blyth A, Daffu-O'Reilly A, Ford J, Hughes CM, Maskrey V, Millar A, Myint PK, Poland FM, Shepstone L, Zermansky A, Holland R, Wright D; CHIPPS Team. The Care Home Independent Prescribing Pharmacist Study (CHIPPS)-a non-randomised feasibility study of independent pharmacist prescribing in care homes. Pilot Feasibility Stud. 2019 Jul 11;5:89. doi: 10.1186/s40814-019-0465-y. PMID: 31338204; PMCID: PMC6625047. | Wrong setting |
| Indar, I., Sampurno, S., Samriah, S., Arifin, A., Mallongi, A., Abadi, Y., Nurhayani, N. The Legal Responsibility of the Doctor on the Family’s Demand to Stop the Treatment of the Terminal Patient that Causes Death. Macedonian Journal of Medical Sciences 2020. | Wrong setting |
| Jansen B, Hughes C, Parsons C. Goals of care in communication between healthcare professionals and caregivers regarding the use of medicines in people with advanced dementia in end of life care. International Journal of Pharmacy Practice. 2012;20(S2):11-12. doi: 10.1111/j.2042-7174.2012.00234_2.x | Wrong publication type |
| Jäger C, Freund T, Steinhäuser J, Stock C, Krisam J, Kaufmann-Kolle P, Wensing M, Szecsenyi J. Impact of a tailored program on the implementation of evidence-based recommendations for multimorbid patients with polypharmacy in primary care practices-results of a cluster-randomized controlled trial. Implement Sci. 2017 Jan 13;12(1):8. doi: 10.1186/s13012-016-0535-y. PMID: 28086976; PMCID: PMC5237147. | Wrong population |
| Jäger C, Steinhäuser J, Freund T, Kuse S, Szecsenyi J, Wensing M. A tailored programme to implement recommendations for multimorbid patients with polypharmacy in primary care practices-process evaluation of a cluster randomized trial. Implement Sci. 2017 Mar 6;12(1):31. doi: 10.1186/s13012-017-0559-y. PMID: 28264693; PMCID: PMC5339959. | Wrong population |
| Jäger C, Szecsenyi J, Steinhäuser J. Design and delivery of a tailored intervention to implement recommendations for multimorbid patients receiving polypharmacy into primary care practices. Biomed Res Int. 2015;2015:938069. doi: 10.1155/2015/938069. Epub 2015 Jan 5. PMID: 25685818; PMCID: PMC4313053. | Wrong population |
| Johnson, M.J., Sheard, L., Noble, S., Prout, H., Maraveyas, A., Watt, I., Dowding, D. To be or not to be? Primary thromboprophylaxis in people with advanced cancer: Views of oncologists and palliative physicians. Palliative Med. - Volume 26, Issue 4, pp. 522 - published 2012-01-01. | Wrong publication type |
| Jokanovic, N., Wang, K.N., Dooley, M.J., Lalic, S., Tan, E.C., Kirkpatrick, C.M., Bell, J.S. Prioritizing interventions to manage polypharmacy in Australian aged care facilities. Res Social Adm Pharm - Volume 13, Issue 3, pp. 564-574 - published 2017-01-01 | Wrong setting |
| Jordan M, Mullan J, Stewart A, Chen TF. A pharmacist integrated into a general practice in Australia: an evolving model of care in medicines optimization. Int J Pharm Pract. 2023 Dec 19;31(6):608-616. doi: 10.1093/ijpp/riad061. PMID: 37823732. | Wrong population |
| Joyce BT, Lau DT. Hospice experiences and approaches to support and assess family caregivers in managing medications for home hospice patients: a providers survey. Palliat Med. 2013 Apr;27(4):329-38. doi: 10.1177/0269216312465650. Epub 2012 Nov 21. PMID: 23175509; PMCID: PMC3606683. | Wrong phenomena of interest |
| Jungo KT, Deml MJ, Schalbetter F, Moor J, Feller M, Lüthold RV, Huibers CJA, Sallevelt BTGM, Meulendijk MC, Spruit M, Schwenkglenks M, Rodondi N, Streit S. A mixed methods analysis of the medication review intervention centered around the use of the 'Systematic Tool to Reduce Inappropriate Prescribing' Assistant (STRIPA) in Swiss primary care practices. BMC Health Serv Res. 2024 Mar 18;24(1):350. doi: 10.1186/s12913-024-10773-y. Erratum in: BMC Health Serv Res. 2024 Apr 2;24(1):413. doi: 10.1186/s12913-024-10896-2. PMID: 38500163; PMCID: PMC10949561. | Wrong population |
| Jungo KT, Mantelli S, Rozsnyai Z et al. General practitioners' deprescribing decisions in older adults with polypharmacy: a case vignette study in 31 countries. BMC Geriatr. 2021 Jan 7;21(1):19. doi: 10.1186/s12877-020-01953-6. PMID: 33413142; PMCID: PMC7792080. | Wrong population |
| Jungo, K.T., Schalbetter, F., Deml, M.J., Streit, S. A mixed methods analysis of the use of the systematic tool to reduce inappropriate prescribing in Swiss primary care practices. Pharmacoepidemiol. Drug Saf. - Volume 29, Issue 0, pp. 145-146 - published 2020-01-01 | Wrong publication type |
| Jungo KT, Weir KR, Cateau D, Streit S. Older adults' attitudes towards deprescribing and medication changes: a longitudinal sub-study of a cluster randomised controlled trial. BMJ Open. 2024 Jan 10;14(1):e075325. doi: 10.1136/bmjopen-2023-075325. PMID: 38199626; PMCID: PMC10806786. | Wrong population |
| Kari H, Kortejärvi H, Laaksonen R. Developing an interprofessional people-centred care model for home-living older people with multimorbidities in a primary care health centre: A community-based study. Explor Res Clin Soc Pharm. 2022 Feb 5;5:100114. doi: 10.1016/j.rcsop.2022.100114. PMID: 35478508; PMCID: PMC9030719. | Wrong population |
| Kerns JW, Winter JD, Winter KM, Kerns CC, Etz RS. Caregiver Perspectives About Using Antipsychotics and Other Medications for Symptoms of Dementia. Gerontologist. 2018 Mar 19;58(2):e35-e45. doi: 10.1093/geront/gnx042. PMID: 28402533. | Wrong population |
| Khuang Lim, C., Miller, C., Jones, T. Promoting advance care planning of patients with parkinsonism in thecommunity. Age Ageing - Volume 50, Issue 0, pp. - published 2021-01-01 | Wrong publication type |
| Kirwan C, Hynes L, Hart N, Mulligan S et al. The multimorbidity collaborative medication review and decision making (MyComrade) study: a pilot cluster randomised trial in two healthcare systems. Pilot Feasibility Stud. 2022 Oct 4;8(1):225. doi: 10.1186/s40814-022-01107-y. PMID: 36195963; PMCID: PMC9531225. | Wrong population |
| Kolovetsios, M., Yones, H. THE ROLE AND IMPACT OF PHARMACISTS WITHIN A HOSPICE'S CARE HOME SUPPORT TEAM. BMJ Support. Palliat. Care - Volume 8, Issue 0, pp. A83-A84 - published 2018-01-01 | Wrong publication type |
| Kuberska K, Scheibl F, Sinnott C, Sheppard JP, Lown M, Williams M, Payne RA, Mant J, McManus RJ, Burt J. GPs' mindlines on deprescribing antihypertensives in older patients with multimorbidity: a qualitative study in English general practice. Br J Gen Pract. 2021 Jun 24;71(708):e498-e507. doi: 10.3399/bjgp21X714305. PMID: 34001537; PMCID: PMC8249009. | Wrong population |
| Latimer A, Pope ND, McFarlin JM. "I Just Feel Like I Always Did": Inotropic Dependency at End of Life. Am J Hosp Palliat Care. 2020 Jul;37(7):497-502. doi: 10.1177/1049909119886302. Epub 2019 Nov 12. PMID: 31714150. | Wrong setting |
| Latter S, Hopkinson JB, Lowson E, Hughes JA, Hughes J, Duke S, Anstey S, Bennett MI, May C, Smith P, Richardson A. Supporting carers to manage pain medication in cancer patients at the end of life: A feasibility trial. Palliat Med. 2018 Jan;32(1):246-256. doi: 10.1177/0269216317715197. Epub 2017 Jul 5. PMID: 28679073. | Wrong phenomena of interest |
| Lau DT, Berman R, Halpern L, Pickard AS, Schrauf R, Witt W. Exploring factors that influence informal caregiving in medication management for home hospice patients. J Palliat Med. 2010 Sep;13(9):1085-90. doi: 10.1089/jpm.2010.0082. PMID: 20836633; PMCID: PMC2964363. | Wrong population |
| Lau DT, Masin-Peters J, Berdes C, Ong M. Perceived barriers that impede provider relations and medication delivery: hospice providers' experiences in nursing homes and private homes. J Palliat Med. 2010 Mar;13(3):305-10. doi: 10.1089/jpm.2009.0283. PMID: 20078242; PMCID: PMC2828533. | Wrong phenomena of interest |
| Lauffenburger JC, Maihan B. Vu, Burkhart, Weinberger, Roth. Design of a Medication Therapy Management Program for Medicare Beneficiaries: Qualitative Findings From Patients and Physicians, The American Journal of Geriatric Pharmacotherapy, Volume 10, Issue 2,  2012, Pages 129-138, ISSN 1543-5946, https://doi.org/10.1016/j.amjopharm.2012.01.002. | Wrong population |
| Laursen J, Kornholt J, Betzer C, Petersen TS, Christensen MB. General Practitioners' Barriers Toward Medication Reviews in Polymedicated Multimorbid Patients: How can a Focus on the Pharmacotherapy in an Outpatient Clinic Support GPs? Health Serv Res Manag Epidemiol. 2018 Sep 14;5:2333392818792169. doi: 10.1177/2333392818792169. PMID: 30246058; PMCID: PMC6144514. | Wrong population |
| Lavan AH, Gallagher P, Parsons C, O'Mahony D. STOPPFrail (Screening Tool of Older Persons Prescriptions in Frail adults with limited life expectancy): consensus validation. Age Ageing. 2017 Jul 1;46(4):600-607. doi: 10.1093/ageing/afx005. PMID: 28119312. | Wrong publication type |
| Low CE, Sanchez Pellecer DE, Santivasi WL, Thompson VH, Elwood T, Davidson AJ, Tlusty JA, Feely MA, Ingram C. Deprescribing in Hospice Patients: Discontinuing Aspirin, Multivitamins, and Statins. Mayo Clin Proc Innov Qual Outcomes. 2021 Jul 24;5(4):721-726. doi: 10.1016/j.mayocpiqo.2021.06.010. PMID: 34355129; PMCID: PMC8325098. | Wrong phenomena of interest |
| Lüthold RV, Cateau D, Jenkinson SP, Streit S, Jungo KT. Pharmacists' attitudes towards interprofessional collaboration to optimise medication use in older patients in Switzerland: a survey study. BMC Health Serv Res. 2024 Jul 26;24(1):849. doi: 10.1186/s12913-024-11339-8. PMID: 39061037; PMCID: PMC11282592. | Wrong population |
| Lyon ME et al. Longitudinal Impact of FAmily CEntered (FACE) Advance Care Planning (ACP) Communication on Willingness to Limit Treatments among Adults Living with HIV/AIDS: A Randomized Clinical Trial. Journal of Pain and Symptom Management, Volume 56, Issue 6, e97 | Wrong publication type |
| MacRobbie et al. Palliative care patient perceptions and experiences of medicines information communication in a community pharmacy: A grounded theory approach. Palliative Med. - Volume 30, Issue 4, pp. S31-S32 - published 2016-01-01 | Wrong publication type |
| Maidment I, Booth A, Mullan J, McKeown J, Bailey S, Wong G. Developing a framework for a novel multi-disciplinary, multi-agency intervention(s), to improve medication management in community-dwelling older people on complex medication regimens (MEMORABLE)--a realist synthesis. Syst Rev. 2017 Jul 3;6(1):125. doi: 10.1186/s13643-017-0528-1. PMID: 28673335; PMCID: PMC5496371. | Wrong publication type |
| Maidment, I.D., Lawson, S., Booth, A., Mullan, J.R., Bailey, S., McKeown, J., Zaman, H., Wong, G. MEMORABLE: MEDICATION MANAGEMENT IN OLDER PEOPLE—REALIST APPROACHES BASED ON LITERATURE AND EVALUATION. Alzheimer's Dementia - Volume 14, Issue 7, pp. P953 - published 2018-01-01 | Wrong publication type |
| Malham CB, Khatib SE, Strumia M, Andrieu S, Cestac P, Salameh P. The MGPIDP-L project: Potentially inappropriate drug prescribing and its associated factors. Arch Gerontol Geriatr. 2023 Jun;109:104947. doi: 10.1016/j.archger.2023.104947. Epub 2023 Jan 28. PMID: 36746016. | Wrong population |
| Mannheimer S, Bergqvist M, Bastholm-Rahmner P, Gustafsson LL, Vég A, Schmidt-Mende K. Experiences of patients receiving Home Care and living with polypharmacy: a qualitative interview study. BJGP Open. 2022 Aug 30;6(2):BJGPO.2021.0181. doi: 10.3399/BJGPO.2021.0181. PMID: 35135816; PMCID: PMC9447318. | Wrong population |
| Mantelli S, Jungo KT, Rozsnyai Z, Reeve E, Luymes CH, Poortvliet RKE, Chiolero A, Rodondi N, Gussekloo J, Streit S. How general practitioners would deprescribe in frail oldest-old with polypharmacy - the LESS study. BMC Fam Pract. 2018 Oct 12;19(1):169. doi: 10.1186/s12875-018-0856-9. PMID: 30314468; PMCID: PMC6186124. | Wrong setting |
| Marcoux, I., Arseneault, C., Blanchette, A., Toupin, M., Youssef, J., Boivin, A. “Medical aid in dying”: Healthcare professionals' comprehension of a possible new practice at the end-of-life. Palliative Med. - Volume 28, Issue 6, pp. 559 - published 2014-01-01. | Wrong publication type |
| Marques, I., Petty, D., Raynor, T., Blenkinsopp, A. Reducing inappropriate polypharmacy through a patient-centred, shared decision-making model of deprescribing. Pharmacoepidemiol. Drug Saf. - Volume 26, Issue 0, pp. 12-13 - published 2017-01-01 | Wrong publication type |
| Martens ESL, Becker D, Abele C, Abbel D, Achterberg WP, Bax JJ, Bertoletti L, Edwards ME, Font C, Gava A, Goedegebuur J, Højen AA, Huisman MV, Kruip MJHA, Mahé I, Mooijaart SP, Pearson M, Seddon K, Szmit S, Noble SIR, Klok FA, Konstantinides SV. Understanding European patterns of deprescribing antithrombotic medication during end-of-life care in patients with cancer. Thromb Res. 2025 Jan;245:109205. doi: 10.1016/j.thromres.2024.109205. Epub 2024 Nov 9. PMID: 39667100. | Wrong setting |
| McCarthy C, Clyne B, Corrigan D, Boland F, Wallace E, Moriarty F, Fahey T, Hughes C, Gillespie P, Smith SM. Supporting prescribing in older people with multimorbidity and significant polypharmacy in primary care (SPPiRE): a cluster randomised controlled trial protocol and pilot. Implement Sci. 2017 Aug 1;12(1):99. doi: 10.1186/s13012-017-0629-1. PMID: 28764753; PMCID: PMC5539883. | Wrong population |
| McCarthy C, Pericin I, Smith SM, Kiely B, Moriarty F, Wallace E, Clyne B. Patient and general practitioner experiences of implementing a medication review intervention in older people with multimorbidity: Process evaluation of the SPPiRE trial. Health Expect. 2022 Dec;25(6):3225-3237. doi: 10.1111/hex.13630. Epub 2022 Oct 17. PMID: 36245339; PMCID: PMC9700182. | Wrong population |
| McGlade, C., Timmons, S., Coffey, A., Weathers, E., Pope, G., Goggin, C., Molloy, W. The influence of healthcare professional's confidence and training in palliative care and treatment choices at the end-of-life. Ir. J. Med. Sci. - Volume 181, Issue 0, pp. S292 - published 2012-01-01 | Wrong publication type |
| McLennon, S., Bakas, T., Jessup, N.M. Stroke family caregiver perceptions of palliative and end of life care needs. Stroke - Volume 46, Issue 0, pp. - published 2015-01-01 | Wrong publication type |
| McPherson, M.L., Cestone, C., Kulo, V., Jun, H.-J., Gordes, K., Stanfield, A.K., Lau, D.T. Learning Outcomes with Infographics vs. Text: Educating Informal Caregivers about Medications. J. Pain Symptom Manage. - Volume 67, Issue 5, pp. e527 - published 2024-01-01 | Wrong publication type |
| Mehdizadeh, D., Johnson, O., Petty, D., Faisal, M., Gardner, P. USING RISK PREDICTION TO CASE-FIND FRAIL OLDER PEOPLE AT RISK OF ANTICHOLINERGIC BURDEN FOR STRUCTURED MEDICATION REVIEWS: A QUALITATIVE STUDY EXPLORING THE VIEWS AND PERSPECTIVES OF PRIMARY CARE PROFESSIONALS. nt. J. Pharm. Pract. - Volume 30, Issue 0, pp. i4 - published 2022-01-01 | Wrong publication type |
| Meijers, J., Hollanders, L., Dijkstra, L., Knapen, E., Dominicus, W., De Weerd-Spaetgens, C., Warmerdam, F., Whichmann, A., Groenewoud, SDesired care in end of life a proactive transmural palliative care pathway. Palliative Med. - Volume 32, Issue 1, pp. 229-230 - published 2018-01-01 | Wrong publication type |
| Mirk, A., Kemp, L., Echt, K.V., Perkins., M.M. Integrated management and polypharmacy review of vulnerable elders: Can we improve outcomes?  J. Am. Geriatr. Soc. - Volume 61, Issue 0, pp. S92 - published 2013-01-01 | Wrong publication type |
| Mitchell GK, Senior HE, Bibo MP, Makoni B, Young SN, Rosenberg JP, Yates P. Evaluation of a pilot of nurse practitioner led, GP supported rural palliative care provision. BMC Palliat Care. 2016 Nov 9;15(1):93. doi: 10.1186/s12904-016-0163-y. PMID: 27829425; PMCID: PMC5103592. | Wrong phenomena of interest |
| Mortier F, Deliens L, Bilsen J, Cosyns M, Ingels K, Vander Stichele R. End-of-life decisions of physicians in the city of Hasselt (Flanders, Belgium). Bioethics. 2000 Jul;14(3):254-67. doi: 10.1111/1467-8519.00195. PMID: 11658137. | Wrong phenomena of interest |
| Mortsiefer A, Löscher S, Pashutina Y, Santos S, Altiner A, Drewelow E, Ritzke M, Wollny A, Thürmann P, Bencheva V, Gogolin M, Meyer G, Abraham J, Fleischer S, Icks A, Montalbo J, Wiese B, Wilm S, Feldmeier G. Family Conferences to Facilitate Deprescribing in Older Outpatients With Frailty and With Polypharmacy: The COFRAIL Cluster Randomized Trial. JAMA Netw Open. 2023 Mar 1;6(3):e234723. doi: 10.1001/jamanetworkopen.2023.4723. PMID: 36972052; PMCID: PMC10043750. | Wrong population |
| Muth C, Harder S, Uhlmann L, Rochon J, Fullerton B, Güthlin C, Erler A, Beyer M, van den Akker M, Perera R, Knottnerus A, Valderas JM, Gerlach FM, Haefeli WE. Pilot study to test the feasibility of a trial design and complex intervention on PRIoritising MUltimedication in Multimorbidity in general practices (PRIMUMpilot). BMJ Open. 2016 Jul 25;6(7):e011613. doi: 10.1136/bmjopen-2016-011613. PMID: 27456328; PMCID: PMC4964238. | Wrong population |
| Neville TH, Tarn DM, Pavlish CL, Wenger NS. The Community Perspective on Potentially Inappropriate Treatment. Ann Am Thorac Soc. 2020 Jul;17(7):854-859. doi: 10.1513/AnnalsATS.201912-890OC. PMID: 32348687; PMCID: PMC7328181. | Wrong setting |
| Niehoff, K., Rajeevan, N., Charpentier, P., Miller, P., Fried., T. Integration of the electronic health record and a clinical decision support system for addressing high-risk medications in older adults. J. Am. Geriatr. Soc. - Volume 63, Issue 0, pp. S207-S208 - published 2015-01-01 | Wrong publication type |
| Niznik JD, Kelley CJ, Fasth L, Colón-Emeric C, Thorpe CT, Gilliam MA, Lund JL, Hanson LC. Deprescribing bisphosphonates for older adults with dementia: perspectives of caregivers. Osteoporos Int. 2024 Apr;35(4):589-598. doi: 10.1007/s00198-023-06967-5. Epub 2023 Nov 18. PMID: 37980284; PMCID: PMC11145563. | Wrong population |
| Norton SA, Wittink MN, Duberstein PR, Prigerson HG, Stanek S, Epstein RM. Family caregiver descriptions of stopping chemotherapy and end-of-life transitions. Support Care Cancer. 2019 Feb;27(2):669-675. doi: 10.1007/s00520-018-4365-0. Epub 2018 Jul 28. PMID: 30056528; PMCID: PMC6338326. | Wrong setting |
| Nottingham, R.P., Kieft, G.V., Murphy, K. Pharmacy services in an outpatient palliative care clinic. J. Palliative Med. - Volume 20, Issue 4, pp. A28 - published 2017-01-01 | Wrong publication type |
| Nyrop KA, Zimmerman S, Sloane PD. Physician perspectives on fall prevention and monitoring in assisted living: a pilot study. J Am Med Dir Assoc. 2011 Jul;12(6):445-50. doi: 10.1016/j.jamda.2010.09.008. Epub 2010 Nov 25. PMID: 21450213. | Wrong publication type |
| O'Caoimh R, Cornally N, McGlade C, Gao Y, O'Herlihy E, Svendrovski A, Clarnette R, Lavan AH, Gallagher P, William Molloy D. Reducing inappropriate prescribing for older adults with advanced frailty: A review based on a survey of practice in four countries. Maturitas. 2019 Aug;126:1-10. doi: 10.1016/j.maturitas.2019.04.212. Epub 2019 Apr 20. PMID: 31239110. | Wrong setting |
| O'Caoimh, Rónán & Cornally, Nicola & O'Herlihy, Eileen & Gao, Yang & Cronin, Una & Coveney, Sarah & Clarnette, Roger & Mcglade, Ciara & Molloy, David. (2014). Opinions Towards a Consensus on Use of Medications in Advanced Dementia. Irish Journal of Medical Science. 183. S312-S312. | Wrong publication type |
| O'Regan, G. & Chipping, S. & Rayner-Barton, J. & Relaeng, Dineo & Davies, D. & Beattie, J.. (2015). The multidisciplinary forum: what do heart failure nurses bring to the table?. European Journal of Cardiovascular Nursing. 14. S73-S73. | Wrong publication type |
| Ouellet, G.M., Hajduk, A., Costello, D., Tinetti, M. Clinicians' perspectives on anticoagulation for atrial fibrillation in persons with dementia. J. Am. Geriatr. Soc. - Volume 68, Issue 0, pp. S152-S153 - published 2020-01-01 | Wrong publication type |
| Ouellet, J., Romegialli, A., Mecca, M.C., Walke, L.M., Kumar, C. Multidisciplinary outpatient geriatrics educational pilot for internal medicine residents. J. Am. Geriatr. Soc. - Volume 65, Issue 0, pp. S124 - published 2017-01-01 | Wrong publication type |
| Pacini M, Smith RD, Wilson EC, Holland R. Home-based medication review in older people: is it cost effective?. Pharmacoeconomics. 2007 Feb;25:171-80. | Wrong population |
| Pang R, Van Leur P, Sullivan S, Wotherspoon C. THE CLINICAL AND PSYCHOSOCIAL IMPACTS ON MANAGEMENT TO PATIENTS WITH COPD IN HARP SETTINGS. InRESPIROLOGY 2016 Nov 1 (Vol. 21, pp. 54-54). 111 RIVER ST, HOBOKEN 07030-5774, NJ USA: WILEY-BLACKWELL. | Wrong publication type |
| Parsons C, Gamble S. Withdrawing antidementia medications in advanced dementia: an analysis of informal caregiver discussions in an online chat forum. International Journal of Pharmacy Practice. 2018 Mar 23;26(S 1):33-4. | Wrong publication type |
| Peat G, Fylan B, Breen L, Raynor DK, Olaniyan J, Alldred DP. Person-centred deprescribing for patients living with frailty: a qualitative interview study and proposal of a collaborative model. Int J Pharm Pract. 2023 May 7;31(3):282-289. doi: 10.1093/ijpp/riad016. PMID: 37068006. | Wrong population |
| Peat G, Fylan B, Marques I, Raynor DK, Breen L, Olaniyan J, Alldred DP. Barriers and facilitators of successful deprescribing as described by older patients living with frailty, their informal carers and clinicians: a qualitative interview study. BMJ Open. 2022 Mar 28;12(3):e054279. doi: 10.1136/bmjopen-2021-054279. PMID: 35351709; PMCID: PMC8961139. | Wrong population |
| Pereira A, Veríssimo M, Ribeiro O. Influence of chronic medical conditions on older patients' willingness to deprescribe medications: a cross-sectional study. BMC Geriatr. 2024 Apr 4;24(1):315. doi: 10.1186/s12877-024-04891-9. PMID: 38575904; PMCID: PMC10993447. | Wrong population |
| Phillips SC, Kamenker-Orlov Y, Suskin D, Schwartz AW. Geriatric 5Ms for Primary Care Residents: A Needs Assessment and Pilot Workshop. InJOURNAL OF THE AMERICAN GERIATRICS SOCIETY 2018 Apr 1 (Vol. 66, pp. S82-S83). 111 RIVER ST, HOBOKEN 07030-5774, NJ USA: WILEY. | Wrong publication type |
| Pickering AN, Hamm ME, Dawdani A, Hanlon JT, Thorpe CT, Gellad WF, Radomski TR. Older patient and caregiver perspectives on medication value and deprescribing: a qualitative study. Journal of the American Geriatrics Society. 2020 Apr;68(4):746-53. | Wrong population |
| Ponton R, Sawyer R. Opioid prescribing in general practice: use of a two-stage review tool to identify and assess high-dose prescribing. Br J Pain. 2018 Aug;12(3):171-182. doi: 10.1177/2049463717742420. Epub 2017 Nov 15. PMID: 30057762; PMCID: PMC6058458. | Wrong population |
| Postavaru GI, McDermott H, Munir F, Ahmed T, Fathers D, Nelson D. Challenges related to the cancer care role. InPSYCHO-ONCOLOGY 2019 Feb 1 (Vol. 28, pp. 19-19). 111 RIVER ST, HOBOKEN 07030-5774, NJ USA: WILEY. | Wrong publication type |
| Previdoli G, Alldred DP, Silcock J, Tyndale-Biscoe S, Okeowo D, Cheong VL, Fylan B. 'It's a job to be done'. Managing polypharmacy at home: A qualitative interview study exploring the experiences of older people living with frailty. Health Expect. 2024 Feb;27(1):e13952. doi: 10.1111/hex.13952. PMID: 39102701; PMCID: PMC10777610. | Wrong population |
| Pruskowski J, Handler S. The Discussion to Ensure the Patient-Centered, Health-Focused, Prognosis-Appropriate, and Rational Medication Regimen (DE-PHARM) Quality Improvement Project—Rational Deprescribing in Residents with Life-Limiting Illness (FR420A). Journal of Pain and Symptom Management. 2017 Feb 1;53(2):359-60. | Wrong publication type |
| Radbruch L, Sabatowski R, Petzke F, Brunsch-Radbruch A, Grond S, Lehmann KA. Transdermal fentanyl for the management of cancer pain: a survey of 1005 patients. Palliative Medicine. 2001 Jun;15(4):309-21. | Wrong phenomena of interest |
| Ragubeer R, Patel HJ. Pilot to improve the appropriate prescription of oral nutritional supplements within the Walsall area. Proceedings of the Nutrition Society. 2011 Jan;70(OCE5):E272. | Wrong publication type |
| Renehan E, Meyer C, Elliott RA, Batchelor F, Said C, Haines T, Goeman D. Posthospital Falls Prevention Intervention: A Mixed-Methods Study. J Aging Phys Act. 2019 Apr 1;27(2):155-165. doi: 10.1123/japa.2017-0406. Epub 2018 Oct 28. PMID: 29989468. | Wrong population |
| Robinson PN, Steel A, Hopwood H, Chindo L, Agbenyegah C, Patel A, Gluck T. Care homes learning together initiative: a collaborative approach to care home medicine. Future Healthcare Journal. 2019 Mar;6(Suppl 1):115. | Wrong publication type |
| Ronquillo C, Stein K. PP102 Developing A Contextually-Informed Deprescribing Intervention. International Journal of Technology Assessment in Health Care. 2018 Jan;34(S1):104-5. | Wrong publication type |
| Rowe S, Pittman N, Balsom C, Druken R, Kelly DV. Beliefs and attitudes of residents, family members and healthcare professionals regarding deprescribing in long-term care: a qualitative study. Int J Clin Pharm. 2022 Dec;44(6):1370-1379. doi: 10.1007/s11096-022-01419-2. Epub 2022 Oct 6. PMID: 36201111. | Wrong setting |
| Rozsnyai Z, Jungo KT, Reeve E, Poortvliet RKE, Rodondi N, Gussekloo J, Streit S. What do older adults with multimorbidity and polypharmacy think about deprescribing? The LESS study - a primary care-based survey. BMC Geriatr. 2020 Oct 31;20(1):435. doi: 10.1186/s12877-020-01843-x. PMID: 33129274; PMCID: PMC7602330. | Wrong population |
| Sadowska K, Turnwald M, O'Neil T, Maust DT, Gerlach LB. Behavioral symptoms and treatment challenges for patients living with dementia: Hospice clinician and caregiver perspectives. J Am Geriatr Soc. 2024 Dec 16. doi: 10.1111/jgs.19320. Epub ahead of print. PMID: 39679782. | Wrong phenomena of interest |
| Sadowski CA, Garrison S, Youngson E, Faulder D, Korownyk T, Bakal J, Kraut R. Pharmacist-led deprescribing of antihypertensive medication in long-term care: the OPTIMIZEBP randomized controlled trial. InINTERNATIONAL JOURNAL OF CLINICAL PHARMACY 2022 Dec 1 (Vol. 44, No. 6, pp. 1563-1563). VAN GODEWIJCKSTRAAT 30, 3311 GZ DORDRECHT, NETHERLANDS: SPRINGER. | Wrong publication type |
| Schäfer I, Kaduszkiewicz H, Mellert C, Löffler C, Mortsiefer A, Ernst A, Stolzenbach CO, Wiese B, Abholz HH, Scherer M, van den Bussche H, Altiner A. Narrative medicine-based intervention in primary care to reduce polypharmacy: results from the cluster-randomised controlled trial MultiCare AGENDA. BMJ Open. 2018 Jan 23;8(1):e017653. doi: 10.1136/bmjopen-2017-017653. PMID: 29362248; PMCID: PMC5786138. | Wrong population |
| Schuling J, Gebben H, Veehof LJ, Haaijer-Ruskamp FM. Deprescribing medication in very elderly patients with multimorbidity: the view of Dutch GPs. A qualitative study. BMC Fam Pract. 2012 Jul 9;13:56. doi: 10.1186/1471-2296-13-56. PMID: 22697490; PMCID: PMC3391990. | Wrong population |
| Sedney C, Kurowski-Burt A, Smith M, Dekeseredy P, Grey C, Boo S. Therapeutic nihilism of neurological diseases: A comparative qualitative study. J Clin Neurosci. 2019 Nov;69:124-131. doi: 10.1016/j.jocn.2019.08.013. Epub 2019 Aug 26. PMID: 31466901. | Wrong setting |
| Sercu M, Beyens I, Cosyns M, Mertens F, Deveugele M, Pype P. Rethinking End-of-Life Care and Palliative Care: Learning From the Illness Trajectories and Lived Experiences of Terminally Ill Patients and Their Family Carers. Qual Health Res. 2018 Dec;28(14):2220-2238. doi: 10.1177/1049732318796477. Epub 2018 Sep 20. PMID: 30234423. | Wrong phenomena of interest |
| Shrestha S, Poudel A, Reeve E, Linsky AM, Steadman KJ, Nissen LM. Development and validation of a tool to understand health care professionals' attitudes towards deprescribing (HATD) in older adults with limited life expectancy. Research in Social and Administrative Pharmacy. 2022 Sep 1;18(9):3596-601. | Wrong phenomena of interest |
| Shega JW, Ellner L, Lau DT, Maxwell TL. Cholinesterase inhibitor and N-methyl-D-aspartic acid receptor antagonist use in older adults with end-stage dementia: a survey of hospice medical directors. Journal of palliative medicine. 2009 Sep 1;12(9):779-83. | Wrong study design |
| Silcock J, Marques I, Olaniyan J, Raynor DK, Baxter H, Gray N, Zaidi STR, Peat G, Fylan B, Breen L, Benn J, Alldred DP. Co-designing an intervention to improve the process of deprescribing for older people living with frailty in the United Kingdom. Health Expect. 2023 Feb;26(1):399-408. doi: 10.1111/hex.13669. Epub 2022 Nov 24. PMID: 36420768; PMCID: PMC9854320. | Wrong population |
| Singier A, Carrier H, Tournier M, Pariente A, Verger P, Salvo F. General practitioners' compliance with benzodiazepine discontinuation guidelines in patients treated with long-term lorazepam: A case-vignette cross-sectional survey. Therapie. 2022 May-Jun;77(3):349-359. doi: 10.1016/j.therap.2021.09.001. Epub 2021 Sep 16. PMID: 34600759. | Wrong population |
| Sinnige J, Korevaar JC, van Lieshout J, Westert GP, Schellevis FG, Braspenning JC. Medication management strategy for older people with polypharmacy in general practice: a qualitative study on prescribing behaviour in primary care. Br J Gen Pract. 2016 Aug;66(649):e540-51. doi: 10.3399/bjgp16X685681. Epub 2016 Jun 6. PMID: 27266862; PMCID: PMC4979928. | Wrong population |
| Sinnott C, Byrne M, Bradley CP. Improving medication management for patients with multimorbidity in primary care: a qualitative feasibility study of the MY COMRADE implementation intervention. Pilot Feasibility Stud. 2017 Mar 20;3:14. doi: 10.1186/s40814-017-0129-8. PMID: 28331631; PMCID: PMC5357807. | Wrong population |
| Sinnott C, Hugh SM, Boyce MB, Bradley CP. What to give the patient who has everything? A qualitative study of prescribing for multimorbidity in primary care. Br J Gen Pract. 2015 Mar;65(632):e184-91. doi: 10.3399/bjgp15X684001. PMID: 25733440; PMCID: PMC4337307 | Wrong population |
| Smucker D, Regan S. Patient safety incidents in home hospice care: a qualitative study of interdisciplinary hospice team members (771). Journal of Pain and Symptom Management. 2012 Feb 1;43(2):462-3. | Wrong publication type |
| Steel A, Hopwood H, Goodwin E, Sampson EL. Multidisciplinary residential home intervention to improve outcomes for frail residents. BMC Health Serv Res. 2022 Jan 12;22(1):58. doi: 10.1186/s12913-021-07407-y. PMID: 35022056; PMCID: PMC8756619. | Wrong setting |
| Stefanacci RG, Reich S, Casiano A. Application of PACE Principles for Population Health Management of Frail Older Adults. Popul Health Manag. 2015 Oct;18(5):367-72. doi: 10.1089/pop.2014.0096. Epub 2015 Feb 3. PMID: 25647691. | Wrong population |
| Sternberg SA, Sabar R, Katz G, Segal R, Fux-Zach L, Grofman V, Roth G, Cohen N, Radomyslaski Z, Bentur N. Home hospice for older people with advanced dementia: a pilot project. Isr J Health Policy Res. 2019 May 6;8(1):42. doi: 10.1186/s13584-019-0304-x. Erratum in: Isr J Health Policy Res. 2019 Jul 1;8(1):56. doi: 10.1186/s13584-019-0329-1. PMID: 31060626; PMCID: PMC6501395. | Wrong phenomena of interest |
| Strandberg TE, Urtamo A, Kähärä J, Strandberg AY, Pitkälä KH, Kautiainen H. Statin Treatment Is Associated With a Neutral Effect on Health-Related Quality of Life Among Community-Dwelling Octogenarian Men: The Helsinki Businessmen Study. J Gerontol A Biol Sci Med Sci. 2018 Sep 11;73(10):1418-1423. doi: 10.1093/gerona/gly073. PMID: 29659717. | Wrong population |
| Subramanian KK, Ramakrishnan V, Rathnam KK. REFUSAL OF PALLIATIVE CHEMOTHERAPY TREATMENT IN ADVANCED LUNG CANCER PATIENTS-PERSPECTIVE FROM A TERTIARY CARE CENTRE IN SOUTH INDIA. InJournal of Thoracic Oncology 2011 Jun 1 (Vol. 6, No. 6, pp. S1488-S1489). 530 WALNUT ST, PHILADELPHIA, PA 19106-3621 USA: LIPPINCOTT WILLIAMS & WILKINS. | Wrong publication type |
| Sun W. A scaling-up approach to educating home care nurses about deprescribing to promote active living of frail older adults at home. InJOURNAL OF THE AMERICAN GERIATRICS SOCIETY 2018 Apr 1 (Vol. 66, pp. S83-S83). 111 RIVER ST, HOBOKEN 07030-5774, NJ USA: WILEY. | Wrong publication type |
| Sun W, Tahsin F, Barakat-Haddad C, Turner JP, Haughian CR, Abbass-Dick J. Exploration of home care nurse's experiences in deprescribing of medications: a qualitative descriptive study. BMJ Open. 2019 May 24;9(5):e025606. doi: 10.1136/bmjopen-2018-025606. PMID: 31129579; PMCID: PMC6538031. | Wrong population |
| Suzuki S, Uchida M, Suga Y, Sugawara H, Kokubun H, Uesawa Y, Nakagawa T, Takase H. A nationwide survey of community pharmacist contributions to polypharmacy in opioid-using and non-using cancer patients in Japan. Biological and Pharmaceutical Bulletin. 2019 Jul 1;42(7):1164-71. | Wrong study design |
| Tait P, Chakraborty A, Jones K, Tieman J. What Is the Community Pharmacists' Role in Supporting Older Australians with Palliative Care Needs? Healthcare (Basel). 2021 Apr 21;9(5):489. doi: 10.3390/healthcare9050489. PMID: 33919247; PMCID: PMC8143164. | Wrong phenomena of interest |
| Tanna N, Ramdoo K, Batten C, Pitkin J, Tatla T. AN INTERFACE COLLABORATIVE RE-DESIGN PROJECT-PREVENTATIVE SCREENING SERVICE FOR COMMUNITY ELDERLY FALLERS. InOSTEOPOROSIS INTERNATIONAL 2016 Nov 1 (Vol. 27, pp. 642-642). 236 GRAYS INN RD, 6TH FLOOR, LONDON WC1X 8HL, ENGLAND: SPRINGER LONDON LTD. | Wrong publication type |
| Thomas C, Biswas R, Hathway C, Collier T, Craven C, Higgins L, Deslandes P. A secondary care led deprescribing approach for care home patients in a health board in Wales. InPHARMACOEPIDEMIOLOGY AND DRUG SAFETY 2017 May 1 (Vol. 26, pp. 17-18). 111 RIVER ST, HOBOKEN 07030-5774, NJ USA: WILEY. | Wrong publication type |
| Tinetti M, Dindo L, Smith CD, Blaum C, Costello D, Ouellet G, Rosen J, Hernandez-Bigos K, Geda M, Naik A. Challenges and strategies in patients’ health priorities-aligned decision-making for older adults with multiple chronic conditions. PloS one. 2019 Jun 10;14(6):e0218249. | Wrong phenomena of interest |
| Turner JP, Edwards S, Stanners M, Shakib S, Bell JS. What factors are important for deprescribing in Australian long-term care facilities? Perspectives of residents and health professionals. BMJ Open. 2016 Mar 10;6(3):e009781. doi: 10.1136/bmjopen-2015-009781. PMID: 26966056; PMCID: PMC4800122. | Wrong setting |
| Ude-Okeleke RC, Aslanpour Z, Dhillon S, Umaru N. Medicines related problems (MRPs) originating in primary care settings in older adults-a systematic review. Journal of Pharmacy Practice. 2023 Apr;36(2):357-69. | Wrong publication type |
| Ude-Okeleke, R.C., Aslanpour, Z., Dhillon, S., Umaru, N. Health Professionals' perception of interventions and strategies to reduce deterioration from Medicines Related Problems in frail older people. Int. J. Pharm. Pract. - Volume 28, Issue 0, pp. 28 - published 2020-01-01 | Wrong publication type |
| van Bruchem-Visser RL, van Dijk G, Mattace Raso F, de Beaufort I. Requests for futile treatments: what mechanisms play a role? Results of a qualitative study among Dutch physicians. BMJ Open. 2020 Apr 28;10(4):e035675. doi: 10.1136/bmjopen-2019-035675. PMID: 32350017; PMCID: PMC7213846. | Wrong phenomena of interest |
| van der Ploeg MA, Streit S, Achterberg WP, Beers E et al. Patient Characteristics and General Practitioners' Advice to Stop Statins in Oldest-Old Patients: a Survey Study Across 30 Countries. J Gen Intern Med. 2019 Sep;34(9):1751-1757. doi: 10.1007/s11606-018-4795-x. Epub 2019 Jan 16. PMID: 30652277; PMCID: PMC6711940. | Wrong phenomena of interest |
| van Summeren JJ, Haaijer-Ruskamp FM, Schuling J. Eliciting Preferences of Multimorbid Elderly Adults in Family Practice Using an Outcome Prioritization Tool. J Am Geriatr Soc. 2016 Nov;64(11):e143-e148. doi: 10.1111/jgs.14415. Epub 2016 Sep 9. PMID: 27612181. | Wrong population |
| Van Der Waal, M.S., Grant, M.P., Uyttewaal, G., Verboeket-Crul, C., Smits-Pelser, H., Teunissen, S.C. Perceptions of Deprescription in Primary Care - A Qualitative Interview Study with Health Care Professionals, Patients and Caregivers. Palliative Med. - Volume 37, Issue 1, pp. 51-52 - published 2023-01-01 | Wrong publication type |
| Walter E, Dawdani A, Hamm ME, Pickering AN, Thorpe CT, Hanlon JT, Gellad W, Radomski TR. PRIMARY CARE PHYSICIANS'PERSPECTIVES ON LOW-VALUE PRESCRIBING IN OLDER ADULTS. InJOURNAL OF GENERAL INTERNAL MEDICINE 2020 Jul 1 (Vol. 35, No. SUPPL 1, pp. S240-S240). ONE NEW YORK PLAZA, SUITE 4600, NEW YORK, NY, UNITED STATES: SPRINGER. | Wrong population |
| Walter EL, Dawdani A, Decker A, Hamm ME, Pickering AN, Hanlon JT, Thorpe CT, Roberts MS, Fine MJ, Gellad WF, Radomski TR. Prescriber perspectives on low‐value prescribing: A qualitative study. Journal of the American Geriatrics Society. 2021 Jun;69(6):1500-7. | Wrong publication type |
| Weber, J.P., Müller, C., Pohontsch, N.J., Böttcher, S., Sekanina, U., Schade, F., Nauck, F., Schneider, N., Scherer, M., Marx, G., Stiel, S. Interaction of General Practitioners and Members of Specialized Outpatient Palliative Care Teams in the Kopal Case Conferences on Patients With Advanced Chronic Diseases: A Qualitative Study. Palliative Med. - Volume 36, Issue 1, pp. 38 - published 2022-01-01 | Wrong publication type |
| Weiss SC, Emanuel LL, Fairclough DL, Emanuel EJ. Understanding the experience of pain in terminally ill patients. Lancet. 2001 Apr 28;357(9265):1311-5. doi: 10.1016/S0140-6736(00)04515-3. PMID: 11343734. | Wrong phenomena of interest |
| Wernli U, Rollin S, Jean-Petit-Matile S, Kobleder A, Meier CR, Meyer-Massetti C. Diabetes management and deprescribing in hospice and palliative care. InINTERNATIONAL JOURNAL OF CLINICAL PHARMACY 2022 Dec 1 (Vol. 44, No. 6, pp. 1496-1497). VAN GODEWIJCKSTRAAT 30, 3311 GZ DORDRECHT, NETHERLANDS: SPRINGER. | Wrong publication type |
| Williams S, Brad LD. Impact of practice based clinical pharmacist led medication reviews on ambulatory patients with hyper polypharmacy. British Journal of General Practice. 2018 Jun 1;68(suppl 1). | Wrong publication type |
| Wilson E, Caswell G, Latif A, Anderson C, Faull C, Pollock K. An exploration of the experiences of professionals supporting patients approaching the end of life in medicines management at home. A qualitative study. BMC Palliat Care. 2020 May 11;19(1):66. doi: 10.1186/s12904-020-0537-z. PMID: 32393231; PMCID: PMC7216477. | Wrong phenomena of interest |
| Wilson E, Caswell G, Pollock K. The 'work' of managing medications when someone is seriously ill and dying at home: A longitudinal qualitative case study of patient and family perspectives'. Palliat Med. 2021 Dec;35(10):1941-1950. doi: 10.1177/02692163211030113. Epub 2021 Jul 12. PMID: 34252329; PMCID: PMC8640265. | Wrong phenomena of interest |
| Yardley, S., Francis, S.-A., Dean Franklin, B., Ogden, M., Kajamaa, A., Mattick, K. Exposing and Understanding the Hidden Work of Getting Prescription Medications Right at Home, in Hospital & Hospice: An Activity Theory Analysis to Improve Patient Safety and Carer Confidence. Palliative Med. - Volume 37, Issue 1, pp. 205-206 - published 2023-01-01 | Wrong publication type |
| Yu SY, Lee YE, Shin SJ, Woo GU, Kim D, Kwon JH, Kim DY, Suh EE. Navigating shared decision-making after the Life-Sustaining Treatment Decision Act: a qualitative study of in-depth interviews with terminal cancer patients, families, and healthcare professionals. Support Care Cancer. 2024 Nov 16;32(12):796. doi: 10.1007/s00520-024-08975-5. PMID: 39549104; PMCID: PMC11568970. | Wrong setting |
| Zanolli NC, Gatta LA, Fish L, Falkovic M, Lorenzo A, Puechl AM, Havrilesky LJ, Davidson B. A Qualitative Assessment of Patient Experience following Systematic Implementation of Goals of Care Conversations in the Ambulatory Gynecologic Oncology Setting. Palliat Med Rep. 2022 Nov 22;3(1):308-315. doi: 10.1089/pmr.2022.0040. PMID: 36479549; PMCID: PMC9712054. | Wrong setting |
| Zechmann S, Trueb C, Valeri F, Streit S, Senn O, Neuner-Jehle S. Barriers and enablers for deprescribing among older, multimorbid patients with polypharmacy: an explorative study from Switzerland. BMC Fam Pract. 2019 May 14;20(1):64. doi: 10.1186/s12875-019-0953-4. PMID: 31088397; PMCID: PMC6518702. | Wrong population |
| Zhong C, Nguyen P, Sung G. End of Life Preferences: Medical Students’ Attitudes Toward Life Support (P5. 9-052). Neurology. 2019 Apr 9;92(15_supplement):P5-9. | Wrong publication type |

**References**

1.	Critical Appraisal Skills Programme (2024). CASP Qualitative Studies Checklist, <https://casp-uk.net/casp-tools-checklists/qualitative-studies-checklist/> (accessed 11 February 2025).

2.	Alwidyan T, McCorry NK and Parsons C. Healthcare professionals’ perspectives of deprescribing in older patients at the end of life in hospice care: a qualitative study using the Theoretical Domains Framework. *International Journal of Pharmacy Practice* 2023; 31: 305-313. DOI: 10.1093/ijpp/riad005.

3.	Cortis L. A qualitative study to describe patient-specific factors that relate to clinical need for and potential to benefit from a medication management service in palliative care. *Journal of Pharmacy Practice and Research* 2017: 34-40. DOI: 10.1002/jppr.1147.

4.	Dees MK, Geijteman ECT, Dekkers WJM, et al. Perspectives of patients, close relatives, nurses, and physicians on end-of-life medication management. *Palliative and Supportive Care* 2018; 16: 580-589. DOI: 10.1017/s1478951517000761.

5.	Geijteman ECT, Dees MK, Tempelman MMA, et al. Understanding the Continuation of Potentially Inappropriate Medications at the End of Life: Perspectives from Individuals and Their Relatives and Physicians. *Journal of the American Geriatrics Society* 2016; 64: 2602-2604. DOI: 10.1111/jgs.14519.

6.	Hall RK, Rutledge J, Lucas A, et al. Stakeholder Perspectives on Factors Related to Deprescribing Potentially Inappropriate Medications in Older Adults Receiving Dialysis. *Clin J Am Soc Nephrol* 2023; 18: 1310-1320. 20230727. DOI: 10.2215/cjn.0000000000000229.

7.	Huisman BAA, Geijteman ECT, Dees MK, et al. Role of nurses in medication management at the end of life: a qualitative interview study. *BMC Palliative Care* 2020; 19. DOI: 10.1186/s12904-020-00574-5.

8.	Kuruvilla L, Weeks G, Eastman P, et al. Medication management for community palliative care patients and the role of a specialist palliative care pharmacist: A qualitative exploration of consumer and health care professional perspectives. *Palliat Med* 2018; 32: 1369-1377. 20180525. DOI: 10.1177/0269216318777390.

9.	Lundby C, Graabæk T, Ryg J, et al. “… Above All, It’s a Matter of  This Person’s Quality of Life”: Health Care Professionals’ Perspectives on Deprescribing in Older Patients With Limited Life Expectancy. *The Gerontologist* 2020; 60: 439-449. DOI: 10.1093/geront/gnz116.

10.	McCloskey B, Hughes C and Parsons C. A qualitative exploration of proxy decision makers' expectations of prescribed medications for people with advanced dementia. *Palliat Med* 2018; 32: 1114-1123. 20180227. DOI: 10.1177/0269216318757163.

11.	Mc Namara KP, Breken BD, Alzubaidi HT, et al. Health professional perspectives on the management of multimorbidity and polypharmacy for older patients in Australia. *Age and Ageing* 2016. DOI: 10.1093/ageing/afw200.

12.	Parsons C and Gamble S. Caregivers’ perspectives and experiences of withdrawing acetylcholinesterase inhibitors and memantine in advanced dementia: a qualitative analysis of an online discussion forum. *BMC Palliative Care* 2019; 18. DOI: 10.1186/s12904-018-0387-0.

13.	Robinson-Barella A, Richardson CL, Bayley Z, et al. “Starting to think that way from the start”: approaching deprescribing decision-making for people accessing palliative care - a qualitative exploration of healthcare professionals views. *BMC Palliative Care* 2024; 23. DOI: 10.1186/s12904-024-01523-2.

14.	Sand AM, Harris J and Rosland JH. Living with advanced cancer and short life expectancy: patients' experiences with managing medication. *J Palliat Care* 2009; 25: 85-91.

15.	Sheard L, Prout H, Dowding D, et al. The ethical decisions UK doctors make regarding advanced cancer patients at the end of life - the perceived (in) appropriateness of anticoagulation for venous thromboembolism: A qualitative study. *BMC Medical Ethics* 2012; 13: 22. DOI: 10.1186/1472-6939-13-22.

16.	Tjia J, DeSanto-Madeya S, Mazor KM, et al. Nurses' Perspectives on Family Caregiver Medication Management Support and Deprescribing. *J Hosp Palliat Nurs* 2019; 21: 312-318. DOI: 10.1097/njh.0000000000000574.

17.	Todd A, Holmes H, Pearson S, et al. ‘I don’t think I’d be frightened if the statins went’: a phenomenological qualitative study exploring medicines use in palliative care patients, carers and healthcare professionals. *BMC Palliative Care* 2016; 15. DOI: 10.1186/s12904-016-0086-7.

18.	Van Der Waal MS, Teunissen SC, Uyttewaal AG, et al. Factors influencing deprescribing in primary care for those towards the end of life: A qualitative interview study with patients and healthcare practitioners. *Palliative Medicine* 2024. DOI: 10.1177/02692163241261202.

19.	Van Middelaar T, Ivens SD, Van Peet PG, et al. Prescribing and deprescribing antihypertensive medication in older people by Dutch general practitioners: a qualitative study. *BMJ Open* 2018; 8: e020871. DOI: 10.1136/bmjopen-2017-020871.

20.	Wyatt K, Bastaki H and Davies N. Delivering end‐of‐life care for patients with cancer at home: Interviews exploring the views and experiences of general practitioners. *Health &amp; Social Care in the Community* 2022; 30. DOI: 10.1111/hsc.13419.

21.	Hong QN, Pluye P, Fàbregues S, et al. Mixed Methods Appraisal Tool (MMAT), version 2018. Registration of Copyright (#1148552), Canadian Intellectual Property Office, Industry Canada. .

22.	Huisman BAA, Geijteman ECT, Kolf N, et al. Physicians' Opinions on Anticoagulant Therapy in Patients with a Limited Life Expectancy. *Seminars in Thrombosis and Hemostasis* 2021; 47: 735-744. DOI: 10.1055/s-0041-1725115.

23.	Huisman BAA, Geijteman ECT, Arevalo JJ, et al. Use of antithrombotics at the end of life: an in-depth chart review study. *BMC Palliative Care* 2021; 20. DOI: 10.1186/s12904-021-00786-3.

24.	Parsons C, McCorry N, Murphy K, et al. Assessment of factors that influence physician decision making regarding medication use in patients with dementia at the end of life. *International Journal of Geriatric Psychiatry* 2014; 29: 281-290. DOI: 10.1002/gps.4006.

25.	Pype P, Mertens F, Helewaut F, et al. Potentially inappropriate medication in primary care at the end of life: a mixed-method study. *Acta Clin Belg* 2018; 73: 213-219. 20171204. DOI: 10.1080/17843286.2017.1410606.
